# Major Novel QTL for Resistance to Cassava Bacterial Blight Identified through a Multi-Environmental Analysis

**DOI:** 10.3389/fpls.2017.01169

**Published:** 2017-07-05

**Authors:** Johana C. Soto Sedano, Rubén E. Mora Moreno, Boby Mathew, Jens Léon, Fabio A. Gómez Cano, Agim Ballvora, Camilo E. López Carrascal

**Affiliations:** ^1^Manihot Biotec Laboratory, Biology Department, Universidad Nacional de ColombiaBogotá, Colombia; ^2^Institute of Crop Science and Resource Conservation-Plant Breeding, University of BonnBonn, Germany

**Keywords:** *Xanthomonas axonopodis* p.v manihotis, candidate defense-related genes, genotype by environment interactions, QTL by environment interaction

## Abstract

Cassava, *Manihot esculenta* Crantz, has been positioned as one of the most promising crops world-wide representing the staple security for more than one billion people mainly in poor countries. Cassava production is constantly threatened by several diseases, including cassava bacterial blight (CBB) caused by *Xanthomonas axonopodis* pv. manihotis (*Xam*), it is the most destructive disease causing heavy yield losses. Here, we report the detection and localization on the genetic map of cassava QTL (Quantitative Trait Loci) conferring resistance to CBB. An F1 mapping population of 117 full sibs was tested for resistance to two *Xam* strains (*Xam*318 and *Xam*681) at two locations in Colombia: La Vega, Cundinamarca and Arauca. The evaluation was conducted in rainy and dry seasons and additional tests were carried out under controlled greenhouse conditions. The phenotypic evaluation of the response to *Xam* revealed continuous variation. Based on composite interval mapping analysis, 5 strain-specific QTL for resistance to *Xam* explaining between 15.8 and 22.1% of phenotypic variance, were detected and localized on a high resolution SNP-based genetic map of cassava. Four of them show stability among the two evaluated seasons. Genotype by environment analysis detected three QTL by environment interactions and the broad sense heritability for *Xam*318 and *Xam*681 were 20 and 53%, respectively. DNA sequence analysis of the QTL intervals revealed 29 candidate defense-related genes (CDRGs), and two of them contain domains related to plant immunity proteins, such as NB-ARC-LRR and WRKY.

## Introduction

Cassava, *Manihot esculenta* Crantz, is a starchy root crop and one of the main staple food crops over the world due to its essential role for food security in tropical regions. This crop represents an important source of calories for about one billion people (Ceballos et al., 2010). Cassava tolerates drought, therefore it has been considered as one of the best alternatives for providing food for the world population in the context of climatic change (Howeler et al., 2013). The major bacterial vascular disease affecting this crop is Cassava Bacterial Blight (CBB), caused by *Xanthomonas axonopodis* pv. manihotis (*Xam*). The disease has a very high destructive power causing losses between 12 and 100% in affected areas (Lozano, 1986; López and Bernal, 2012). *Xam* has been described among the top 10 most important plant pathogenic bacteria (Mansfield et al., 2012). CBB has been reported in all regions where cassava is grown (López and Bernal, 2012; Taylor et al., 2012), including 56 countries distributed over Asia, Africa, Oceania and North, Central, and South America (http://www.cabi.org/) and the number of countries affected by the disease is increasing. The advancement of CBB epidemics has been reported in many countries, with Burkina Faso, being one of the most recent ones (Wonni et al., 2015). The Colombian *Xam* populations remain highly dynamic and exhibit a high genetic diversity (Trujillo et al., 2014). The analysis of 65 *Xam* genomes revealed that this pathogen harbors 14–22 effector genes, from which nine are conserved in all the strains (Bart et al., 2012).

Although some measures such as planting of disease-free material can be applied in the cassava fields in order to protect the crop, the safest and most efficient strategy to control CBB is to take advantage of natural plant genetic resistance to develop resistant cultivars for cultivation in CBB prone regions. Plants have evolved several mechanisms to defend themselves against pathogens. These mechanisms have been extensively studied in model plants, but knowledge generated in cassava is relatively scarce. Histology and cytochemistry studies of the resistance mechanisms in cassava during *Xam* infection showed callose deposition that act as a barrier in cortical parenchyma cells and phloem to block bacterial multiplication and dispersion (Kpémoua et al., 1996; Sandino et al., 2015). Other mechanisms of defense response including cell wall fortification, lignification and suberization associated with callose deposition and production of flavonoids and polysaccharides were also observed and they are faster and stronger in resistant cultivars compared to susceptible ones (Kpémoua et al., 1996). On the other side, efforts have been made in the last years to identify molecular determinants of the CBB resistance, including the selective analysis of homologous genes coding for proteins containing NBS and TIR domains (Lopez et al., 2003) or by annotation of sequence information based on the cassava genome sequence draft (Lozano et al., 2015; Soto et al., 2015).

Resistance to CBB has been described as quantitative, showing polygenic and additive inheritance (Hahn et al., 1974; Jorge et al., 2000, 2001) occurring together with resistance to cassava mosaic disease (CMD) (Hahn et al., 1980; Lokko et al., 2004; Rabbi et al., 2014). Several quantitative trait loci for resistance to CBB have been identified in cassava using full-sib population derived from the cross TMS30572 × CM1477-2. Eight QTL, explaining between 7.2 and 18.2% of the variance were detected in field conditions under high disease pressure and over two consecutive crop cycles (Jorge et al., 2001). Under controlled conditions, 12 resistance QTL were identified to five *Xam* strains, explaining 9–27% of the phenotypic variance (Jorge et al., 2000). Two other QTL were identified for resistance against *Xam* strains CIO151 and CIO121 explaining 62 and 21% resistance, respectively (Lopez et al., 2007). Moreover, Wydra et al. (2004) reported nine QTL explaining from 16 to 33% of the resistance variance to four African *Xam* strains.

The environment plays an important role in the phenotypic variation of quantitative traits (Weinig and Schmitt, 2004; Anderson et al., 2014). Consequently, the detection and stability of the QTL between environments is an important aspect to consider when studying genetic determinants of complex traits (Anderson et al., 2013; Mitchell-Olds, 2013; El-Soda et al., 2014). A QTL by environment interaction (Q × E) is defined as a different expression of the trait in varying environments, like having a significant effect in one environment but not in another (El-Soda et al., 2014). The complexity of the analysis increases when the plant-pathogen interaction under various environmental conditions is studied. According to the classic quantitative genetic model, the phenotype is the result of the genetic composition of the plant (G), the environment (E) and of the interaction between them (GxE). However, genotypes by environment interactions are additionally complicated in the case of plant-pathogen interactions in which another genotype (corresponding to the pathogen) must be considered. In this case, the equation of this form of interaction will result in interaction = G_plant_ × G_pathogen_ × E (Jorgensen, 2012). In this context, the detection of QTL and the dissection of their allelic composition are necessary to better understand the genetic basis of these interactions and the phenotypic responses to specific environmental conditions (El-Soda et al., 2014).

The molecular bases and mechanisms of quantitative resistance in plants are not well known. Interestingly, recent reports, including the cloning of genes operating in QTL, have provided some important clues that would facilitate the unraveling of the molecular bases of quantitative resistance in cassava. The proteins involved in pathogen recognition can be similar in both qualitative and quantitative resistance (Gebhardt and Valkonen, 2001; Lopez et al., 2003; Ramalingam et al., 2003; Calenge and Durel, 2006; Poland et al., 2009; Kou and Wang, 2010; Roux et al., 2014; Corwin et al., 2016). In addition, genes involved in quantitative resistance can correspond to those coding for proteins involved in the signal transduction pathways of defense response (Fukuoka et al., 2009; Corwin et al., 2016) and/or possessing antimicrobial activity (Ramalingam et al., 2003; Guimaraes and Stotz, 2004; Liu et al., 2006; Van Loon et al., 2006).

The aim of this study was to better understand the mechanistic basis of plant response to CBB. By analyzing a segregating population for disease resistance in several locations with various environmental conditions allows not only the identification of genetic factors leading to resistance, but as well the interaction of specific genotype with the environment.

Here, we report the identification of 5 novel QTL for specific resistance against two *Xam* strains in cassava, detected in field evaluations during rainy and dry seasons in two Colombian environments, as well under greenhouse conditions. Furthermore, the analysis of G × E and QTL × E interactions (Q × E), allowed to estimate the impact of the environment over the QTL effect. Candidate defense-related genes (CDRGs) located within the QTL intervals were identified based on the *in silico* analysis of DNA sequence information from the corresponding chromosome regions using whole genome sequence draft of cassava.

## Materials and methods

### Plant material

The mapping population consists of 117 full sib F1 segregating population derived from a cross between Nigerian cultivar TMS30572 and CIAT's elite cultivar CM2177-2 (Fregene et al., 1997). This population has been used extensively in mapping studies (Fregene et al., 1997; Jorge et al., 2000; Mba et al., 2001; Lopez et al., 2007; Soto et al., 2015). To produce multiple clonal plants of each individual, parent and F1 progeny were grown and propagated vegetatively from stem-cuttings in CBB-free field conditions at La Vega (Cundinamarca) and Universidad Nacional de Colombia, at Arauca (Arauca).

### Bacterial strains and phenotypic evaluation of CBB resistance

For long-term storage, bacteria strains *Xam318* and *Xam681* were kept in 60% glycerol at −80°C, and streaked on LPGA medium [yeast extract (5 g/Lt), peptone (5 g/Lt), glucose (5 g/Lt), bacto-agar (15 g/Lt)] at 28°C for 12 h before use as inoculum. A single colony of *Xam* was grown in liquid culture at 28°C with shaking at 230 rpm for 24 h. Cells were harvested by centrifugation at 3,000 g and re-suspended in 10 mM MgCl_2_. Based on a preliminary screen, the *Xam* strains *Xam318* and *Xam681* were selected because the parent TMS30572 is resistant to these two strains while line CM2177-2, is susceptible (data not shown and Trujillo et al., 2014). The *Xam* strains were isolated from plant samples with typical symptoms of CBB disease in fields located at Ciénaga de Oro (N 08.889°, W 075.569°) a typical Caribbean savanna region and Palmitos (N 09.450°, W 075,160°) located on mountains.

For the evaluation of CBB disease response, plants were grown on two different locations: La Vega Cundinamarca, Andina region (Latitude 0,5°,00′44.188″N, Longitude 74°21′31.005″N) and Arauca, Arauca, Orinoquía region (Latitude 7° 1′ 22.32″N, Longitude 70° 44′ 42.50″W). Six-week old plants were inoculated on July 2013 and December 2014, corresponding to rainy and dry seasons, respectively. Besides, an evaluation was done under greenhouse controlled conditions. The corresponding codes for the different conditions, environments, strains and seasons, are presented in Table 1.

**Table 1:** Location, season, temperatures, relative humidity and mean precipitation of the environments, *Xam* strain and code where the inoculation and phenotyping were conducted.

**Location**	**Season**	**Max. Temperature (C°)^a^**	**Min. Temperature (C°)^a^**	**Relative humidity (%)^a^**	**Mean precipitation (mm)^a^**	***Xam* strain**	**Code**
Arauca (Arauca)	Rainy	31	22	88	301	*Xam*318	AR318-R
	Dry	31	22	73	18,7	*Xam*318	AR318-D
	Rainy	31	22	88	301	*Xam*681	AR681-R
	Dry	31	22	73	18,7	*Xam*681	AR681-D
La Vega (Cundinamarca)	Rainy	20	9	79	106	*Xam*318	LV318-R
	Dry	22	10	70	30	*Xam*318	LV318-D
	Rainy	20	9	79	106	*Xam*681	LV681-R
	Dry	22	10	70	30	*Xam*681	LV681-D
Greenhouse 12 h of photoperiod	2013	30	20	70	–	*Xam*318	G318-2013
	2014	30	20	70	–	*Xam*318	G318-2014
	2013	30	20	70	–	*Xam*681	G681-2013
	2014	30	20	70	–	*Xam*681	G681-2014

a *(IDEAM. www.ideam.gov.co)*.

The inoculation was conducted by puncturing the stem of each plant between the second and third true leaf. The bacterial suspension was placed using a tip filled with 10 μl of the inoculum (1 × 10^6^ UFC/mL). As mock, one plant was inoculated with 10 mM of MgCl_2_. The disease severity was scored at 7, 14, 21, and 30 days after inoculation, using a rating from 0 to 5 according to the symptoms scale proposed by Verdier et al. (1994), where 0 = no symptoms, 1= necrosis at the inoculation point, 2= stem exudates, 3 = one or two wilted leaves, 4 = more than three wilted leaves and 5 = plant death. Disease progress in time was estimated for each replicate by calculating the area under disease progress curve (AUDPC) (Shaner and Finney, 1977; Jeger and Viljanen-Rollinson, 2001). Once the phenotyping evaluation was finished, the inoculated material and the substrate (soil) were burned to ensure the CBB-free field condition. In order to determine resistance and susceptibility to the strain in parents and F1 genotypes, the AUDPC value was taken into account as well as the criteria previously described (Jorge et al., 2000; Restrepo et al., 2000; Trujillo et al., 2014). Even though cassava resistance to CBB is quantitative, there are several reports how to define a response as resistant or susceptible. According to Restrepo et al. (2000), if one of five biological replicate shows a scale of symptoms of 4 or 5 at 30 dpi, the genotype is considered as susceptible. On the other hand, Jorge et al. (2000) classify a genotype as susceptible when the average of the symptom using the scale, is greater than or equal to 3. Finally, the criteria described by Trujillo et al. (2014), the cassava genotypes with logAUDPC values lower than 1.59 belong to resistant genotypes while those with AUDPC values higher than 1.69 to susceptible ones.

### Statistical analysis

For each location, including greenhouse, year and strain, five biological replications (clonal plants) per genotype and mock were disposed according to a randomized complete design. A Log transformation for the AUDPC values were done as described by Restrepo et al. (2000). The normality of AUDPC values were tested using Shapiro-Wilkinson test, whereas the correlation coefficients for the phenotypic data between different environments were calculated using the Pearson method. The variance components were estimated using ANOVA. All the above statistical analyses were performed using the R package.

Broad-sense heritability (H^2^) of the response to *Xam* strains was determined by calculating the genetic variance component and the residual or environmental variance component, using lme4 package (Bates et al., 2015) in R package (Ripley, 2001). The variance components of genotype, genotype by environment, genotype by year and experimental error, were measured according to Holland (2006). The value of heterobeltiosis (Jinks and Jones, 1958), was measured in order to establish the percentage of progeny that exhibited higher levels of resistance than the resistant parent. In order to evaluate performance (response to bacteria) of cassava genotypes under CBB incidence, a genotype x environment analysis was performed through a GGE-biplot analysis of multi-environment trials (MET) separately by bacteria strain and year of evaluation (rainy and dry seasons), based on the model for two principal components (Yan et al., 2000; Yan and Tinker, 2006). Data from all locations, including greenhouse, were combined to construct the GGE-biplots in order to compare the behavior of the genotypes. The GGE-biplot criteria were centered by two (centered G + GE), without scaling and singular value partitioning (SVP). GGE-biplot analysis was performed using the R package GGEBiplotGUI (Frutos-Bernal and Galindo, 2012).

### QTL mapping

The QTL mapping analysis was performed with Composite Interval Mapping (CIM) using the Haley–Knott regression approach in R/qtl V1.37-11 (Broman, 2015) by employing three markers as covariate in a window size of 10 cM. The genetic positions of the 2,571 polymorphic heterozygous SNP markers are based on the genetic map distributed on 19 linkage groups with an overall size of 2,571 cM and an average distance of 1.26 cM between markers (Soto et al., 2015). A LOD threshold was calculated using 1,000 permutation tests. The LOD peak of marker-traits associations was taken into account to define the QTL position on the linkage groups in the map. The QTL interval was confined by a LOD decrease value of 1.5 compared to the peak. The phenotypic variation (*R*^2^) explained by each QTL was determined through the function calc.Rsq in R package. Environmental effects to the QTL were estimated by using the significant (LOD 2,5) additive phenotypic effects (APE) of QTL and performed using the software QTL IciMapping (Meng et al., 2015).

### Identification of candidate genes

Candidate genes were located using the information of physical positions of the SNP-based genetic map. BLAST (Basic Local Alignment Search Tool) analysis was conducted to the current cassava genome sequence draft (v6.1) implemented at the JGI's Phytozome platform.

## Results

### Evaluation of CBB resistance in mapping population

The F1 population and parents were evaluated in two different agro-ecological locations (Arauca and La Vega) during two consecutive dry and two rainy seasons. All AUDPC values for the F1 genotypes for each location, *Xam* strain and season showed a continuous and normal distribution, except for LV681-D (Supplementary Material 1). As was expected, the parents exhibited AUDPC values in both extremes of the distribution curve. At the end of the trial the genotype TMS30572 was considered as resistant with AUDPC values ranging from 1.21 for LV318-R, to 1.39 for AR318-R. On the other hand, the susceptible genotype CM2177-2, exhibiting high AUDPC values of 1.72 for AR318-R to 1.92 for LV681-R. The AUDPC values observed among the genotypes were found to be highest in AR318-D (ranged from 0.97 to 1.78), followed by G681-2013 (from 1.08 to 1.89) and AR318-R (from 1.23 to 1.78) in that order. The number of genotypes evaluated, the AUDPC of the parents and the distribution of AUDPC values in the progeny for each location are shown in Table 2.

**Table 2:** Distribution of AUDPC values in the mapping population.

**Location**	**Genotypes evaluated**	**AUDPC CM2177-2**	**AUDPC TMS30572**	**AUDPC range**	**Resistant genotypes**	**Susceptible genotypes**	**Resistant transgressive phenotype**	**Susceptible transgressive phenotype**
AR318-R	103	1.72	1.39	1.23–1.78	79	24	25	4
AR318-D	100	1.76	1.26	0.97–1.78	84	16	11	1
AR681-R	104	1.83	1.35	1.28–1.94	68	36	6	2
AR681-D	100	1.8	1.32	1.16–1.94	72	28	8	3
LV318-R	93	1.85	1.21	1.16–1.82	67	26	3	0
LV318-D	106	1.73	1.24	1.04–1.82	80	26	15	7
LV681-R	93	1.92	1.39	1.19–1.97	55	38	6	2
LV681-D	106	1.88	1.36	1.21–1.97	74	32	12	6
G318-2013	117	1.85	1.28	1.09–1.88	78	39	15	1
G318-2014	109	1.87	1.28	1.10–1.86	73	36	16	0
G681-2013	117	1.87	1.25	1.08–1.89	74	43	18	1
G681-2014	112	1.87	1.25	1.10–1.90	73	39	18	2

*Number of genotypes evaluated, AUDPC values for parents, range of AUDPC values obtained in each location, number of resistant and susceptible genotypes in the population for each location and number of resistant and susceptible transgressive phenotypes by environment. AR, Arauca; LV, La Vega; G, greenhouse; R rainy season; D, dry season; 318, Xam318; 681, Xam681*.

In all the locations and seasons tested, the number of resistant genotypes was higher than that of the susceptible ones for both *Xam* strains (Table 2). Results indicated that more genotypes exhibited resistance to CBB during the dry season compared with the rainy season. About 84 and 75% of the progeny were resistant to *Xam*318 under dry season in Arauca and La Vega, respectively; but were reduced to 77% in Arauca and 72% in La Vega during the rainy season. In dry season, 72 and 70% of the progeny showed resistance against *Xam*681 in Arauca and La Vega, respectively. These values dropped to 65 and 59%, respectively, during the rainy season (Table 2).

In order to detect possible differential response of the genotypes against the pathogen, the phenotypic plasticity was determined. In at least one location and/or season, 48% of the genotypes showed a distinct phenotype for the strain *Xam*318, while 38% were observed for *Xam*681 (Supplementary Material 2). For instance, genotypes g5, g40, g53, and g116 were susceptible in AR318-R but were resistant under all the other conditions tested. On the other hand, genotypes g15, g51, and g97, which showed a resistant response to *Xam*681 in La Vega during rainy and dry seasons, were susceptible under the rainy season in Arauca.

The mapping population exhibited a transgressive segregation for resistance to *Xam* strains. Transgressive genotypes with higher resistance or susceptibility than the parents were identified in the two locations, against the two *Xam* strains and also under rainy and dry seasons (Table 2). However, no statistically significantly differences between the transgressive segregants and its parents were found. Locations LV318-D and LV681-D provided the highest number of transgresses, with seven and six respectively. For all conditions, the total number of resistant transgresses was higher than susceptible ones. The highest number of resistant transgresses was identified at AR318-R with 25 transgresses and in the greenhouse conditions against *Xam*681 in both G681-2013 and G681-2014, with 18 and 18 transgresses each (Table 2). The genotype g79 exhibited a resistant transgressive phenotype in six of the 12 conditions evaluated (Arauca, for both *Xam* strains, and also during rainy and dry seasons). The greenhouse evaluation using *Xam*681 showed lower AUDPC values in relation to the resistant parent. However, the genotype g29 was identified as a susceptible transgressive phenotype for six of the 12 conditions: AR681-R, AR681-D, LV681-D, G318-2013, G681-2013 and G681-2014 (Supplementary Material 2).

Pairwise Pearson correlation between AUDPC values measured at two locations and greenhouse, two seasons and for the two *Xam* strains were highly significant (*P* < 0.05) with correlation coefficients ranging between 0.62 for pairwise comparison of AR318-R and AR318-D to 0.99 for G681-2013 and G681-2014 (Table 3). Nevertheless, very low values of correlation were found between different environments.

**Table 3:** Pairwise Pearson correlation coefficients between AUDPC values for all environments and for the two *Xam* strains employed.

**Location**	**AR318-R**	**AR681-R**	**LV318-R**	**LV681-R**	**G318-2013**	**G681-2013**
AR318-D	**0.62**					
AR681-D	0.06	**0.84**				
LV318-D	0.03	0.08	**0.81**			
LV681-D	0.15	0.35	0.05	**0.89**		
G318-2014	0.07	0.15	0.04	0.05	**0.91**	
G681-2014	−003	0.34	0.04	0.22	0.34	**0.99**

AR, Arauca; LV, La Vega; G, greenhouse; R, rainy season; D, dry season; P-value = 0.05

Broad sense heritability of the resistance to *Xam*318 was 20%, with values of 0.0022 and 0.0110 for genetic and environmental variance, respectively. While the broad sense heritability of the resistance to *Xam*681 was 53%, with values of 0.0075 and 0.0141 for genetic and environmental variance, respectively. The highest and most stable values of heterobeltiosis were observed for the tests conducted under greenhouse conditions. For strain *Xam*681 the values were 21.11 and 20.39% in 2013 and 2014, respectively, whereas for *Xam*318 it was 16.62% for both years of evaluation (Table 4).

**Table 4:** Better-parent heterosis.

	**AR-R (%)**	**AR-D (%)**	**LV-R (%)**	**LV-D (%)**	**G-2013 (%)**	**G-2014 (%)**
*Xam*681	14.36	14.60	13.46	14.15	21.11	20.39
*Xam*318	7.43	14.24	14.60	16.59	16.62	16.62

*Percentage of heterosis over the better parent values for the quantitative genetic trait of resistance to the two Xam strains evaluated in cassava under the environments AR, Arauca; LV, La Vega; G, greenhouse; R, rainy season; D, dry season*.

**Figure 1:**
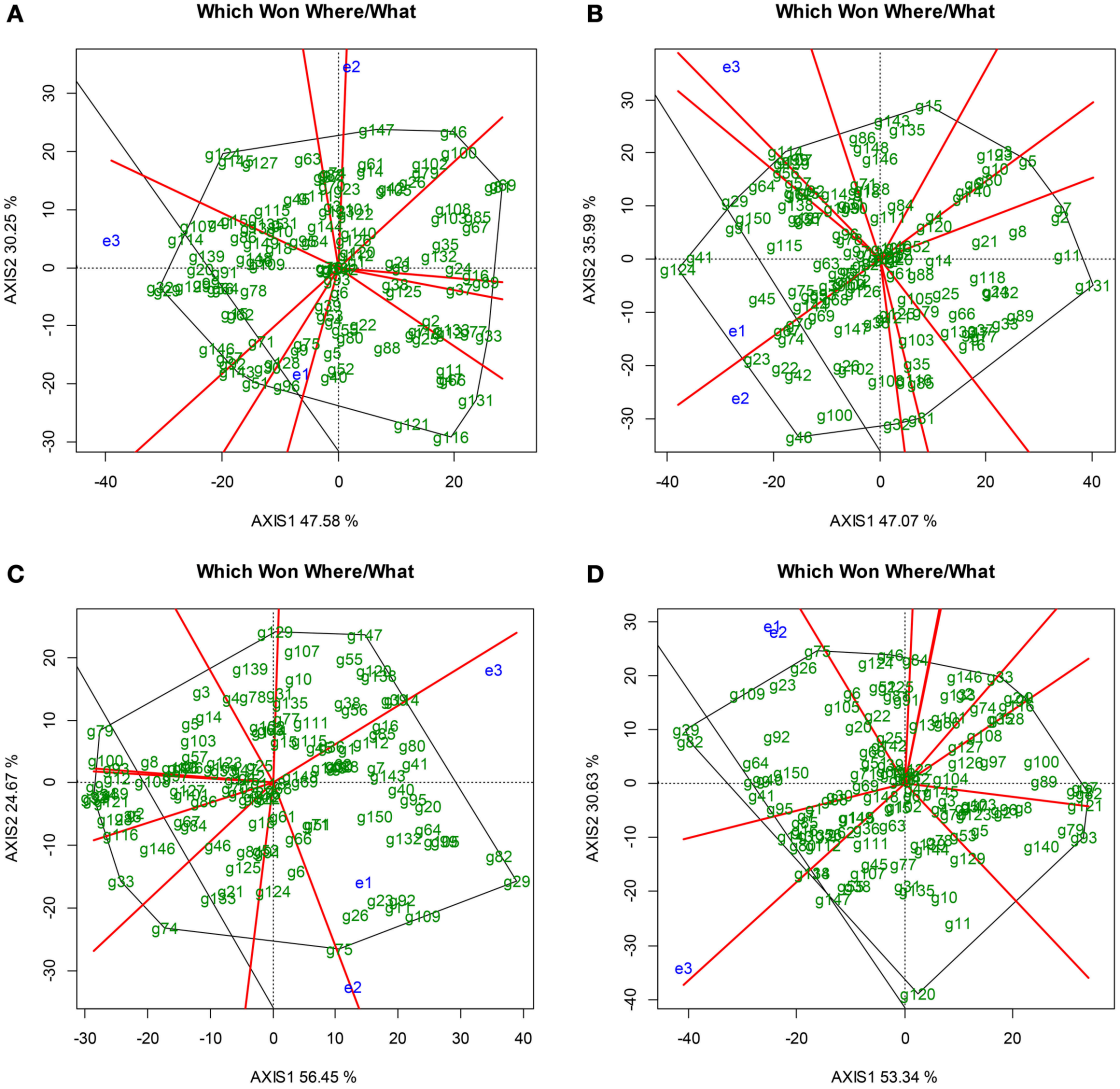
Which Won Where/What graphic of GGE-Biplot analysis. **(A)** GGE-Biplot analysis for phenotype evaluation against *Xam*318 under rainy season. **(B)** GGE-Biplot analysis for phenotype evaluation against *Xam*318 under dry season. **(C)** GGE-Biplot analysis for phenotype evaluation against *Xam*681 during the rainy season. **(D)** GGE-Biplot analysis for phenotype evaluation against *Xam*681 during the dry season. e1, Arauca; e2, La Vega; e3, Greenhouse.

The analysis of variance showed significant differences (*p* < 0.001) for genotype (g), environment (location, season), *Xam* strain, and genotype x environment among the F1 genotypes tested (Supplementary Material 3). The GGE-Biplot analysis revealed that the two principal components explained 77.83% of the total variance caused by G_plant_ + G_pathogen_ + E for cassava resistance to *Xam*318 during rainy season, 83.96% for *Xam*318 during dry season, 81.12% for *Xam*681 in rainy season and 83.97% for *Xam*681 in dry season (Figure 1). Also, the AUDPC values of the genotypes were able to discriminate between environments. The behavior of genotypes differed between environments and these fall into two sectors of the graphic, except for *Xam*318 during the rainy season, which fall in three sectors (Figure 1). In both Arauca and La Vega, except for *Xam*318 rainy season, the genotypes behavior seems to be quite similar (Figures 1B–D).

The environments with short vectors indicate that the genotypes under this environment behave similarly. This is the case for the evaluation in Arauca (e1) against *Xam*318 during rainy and dry season (Figures 1A,B). In the same category falls the evaluation conducted for *Xam*681 in Arauca during rainy season (Figure 1D). On the other side, large vectors such as those exhibited by all the evaluations under the greenhouse condition, indicates higher level of dissimilarity between the phenotypic responses of the genotypes (Figure 1).Using the criteria previously described (Jorge et al., 2000; Restrepo et al., 2000; Trujillo et al., 2014), it was possible to distinguish genotypes as the “extremes” for resistant and susceptible for almost all environments. Genotypes g33 and g41 were selected as extreme resistant and susceptible, respectively in response to *Xam*318 in rainy season. During the dry season, the extreme resistant genotype against the same strain was g131, while g124 was identified as the extreme susceptible genotype. On the other hand, the evaluation conducted under the rainy season employing *Xam*681 allowed to distinguish g79 and g29 as extreme genotypes for resistance and susceptibility, respectively. The genotype g93 was identified as the extreme resistant and g29 as the extreme susceptible in the dry season. The g29 showed to be the most susceptible for the two *Xam* strains and it also had the highest susceptible transgressive phenotype.

### Identification of QTL for resistance to *Xam* in cassava

**Figure 2:**
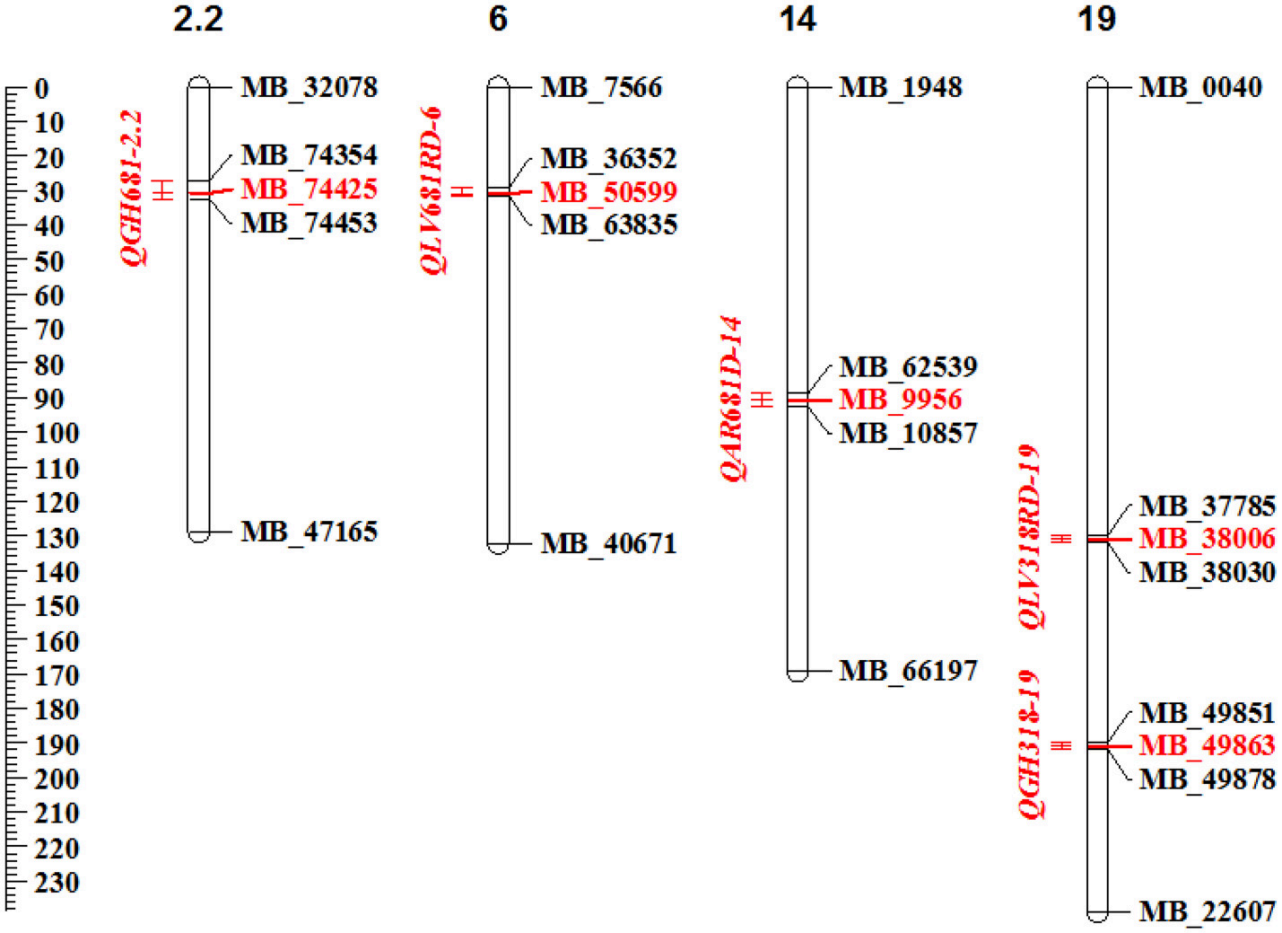
Schematic view of the QTL for resistance to CBB located within cassava linkage groups. In the represented cassava linkage groups are shown the detected QTL, in red letters are shown the QTL peak markers, in black, the flanking markers. The genetic distances in cM are shown with the scale on the left. Diagram plotted using MapChart software (Voorrips, 2002).

In total 5 QTL distributed in 4 of the 19 linkage groups (LG) were detected (Figure 2). The *QGH681-2.2* located on the LG 2.2, the *QLV681RD-6* on the LG 6, the *QAR681D-14* on LG14 and the *QLV318RD-19* and *QGH318-19* located on the LG 19. The QTL detected in the evaluation at La Vega during the rainy season (*QLV681RD-6)* explaining 22.1% of the resistance to *Xam*681, showed the highest LOD value of 5.0 (Table 5). The phenotypic variance of resistance to *Xam* explained by all identified QTL ranged from 15.8 to 22.1%. The evaluation against *Xam*318 detected two QTL, explaining 17.3–18.8% of the phenotypic variance, whereas three QTL were identified for resistance to *Xam*681 accounting for 15.8–22.1% of the phenotypic variance. From the 5 QTL, one was detected at Arauca location, whereas two QTL were identified each at Vega and greenhouse conditions.

**Table 5:** Summary of QTL for resistance to CBB.

**QTL name**	**LOD score**	***R*^2^**	**Peak Marker**	**Pos. cM**	**Marker Interval**	**Interval length cM**	**Interval length Kb**	**Genes in interval**
*QAR681D-14*	**4.3**	18.1%	MB_9956	90.6	MB_62539/MB_10857	4.1	7.1	2
*QLV318RD-19*	**3.8**	17.3%	MB_38006	^*^	MB_37785/MB_38030	^*^	80.8	8
*QLV681RD-6*	**5.0**	22.1%	MB_50599	31.0	MB_36352/MB_63835	2.2	6.5	3
*QGH318-19*	**4.9**	18.8%	MB_49863	^*^	MB_49851/MB_49878	^*^	58.8	8
*QGH681-2.2*	**4.2**	15.8%	MB_23160	93.5	MB_23143/MB_26945	2.3	110.1	8

*QTL name, location, Xam strain and linkage group), LOD threshold, R^2^, percentage of phenotypic variance explained by the QTL, peak marker, position of the peak marker in the genetic map given in cM. QTL marker interval, interval length in cM and Kb and number of genes within the intervals is shown. Underlined stable QTL between seasons for the same location. In bold highly significant QTL based on LOD obtained by permutation test*.

* *Unknown genetic position*.

On average, the interval length of the QTL was 2.8 cM, with the lowest interval length 2.2 cM for *QLV681RD-6* and the largest of 4.1 cM for *QAR681D-14* (Table 5). The QTL *QGH681-2.2, QLV681RD-6, QLV318RD-19*, and *QGH318-19* were stable between the two seasons for the same location. From these, two correspond to QTL detected under greenhouse conditions and two under La Vega conditions. Two of the stable QTL confer resistance to *Xam*681 and the other two to *Xam*318 (Table 5). There were no QTL detected for all environments and/or seasons with LOD score above the threshold.

### Interaction of QTL with environment

**Figure 3:**
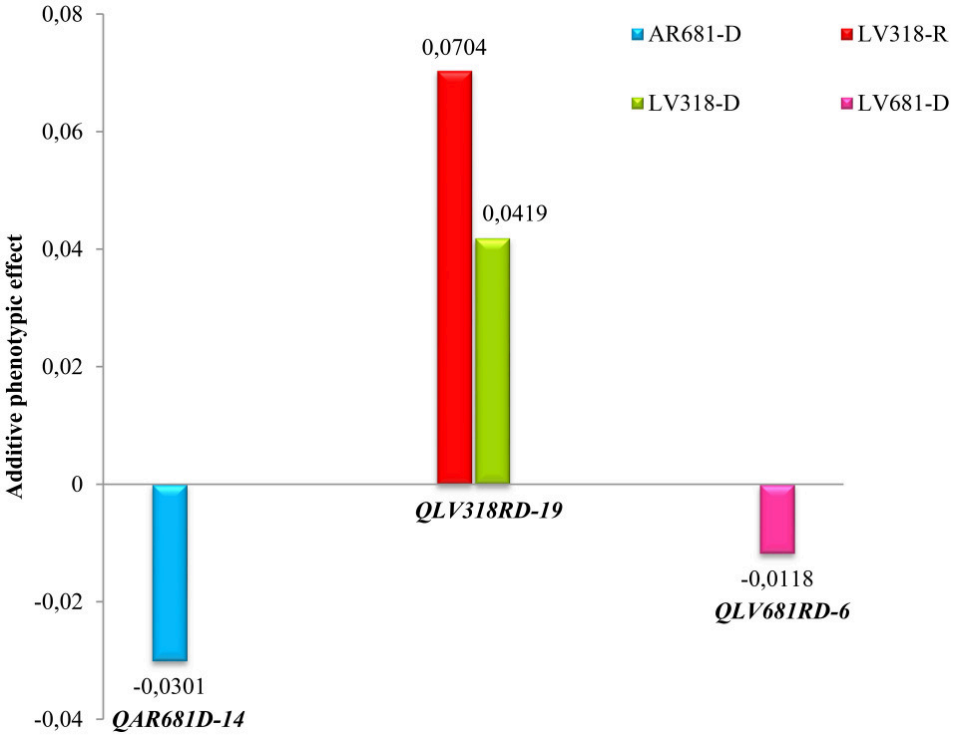
QTL x environment interactions based on additive phenotypic effects (APE). QTL exhibit significant Q x E interactions when considering significant (LOD 2,5) additive phenotypic effects (APE). Positive APE was correlated with susceptibility to *Xam* while negative APE with resistance.

In order to estimate the environment effects on the QTL, the Q × E interaction analysis was conducted. In this case, the APE obtained through the QTL IciMapping was emloyed as a measure of the environment effect on the QTL identification. The QTL detected under greenhouse conditions were not taken into account. Three QTL exhibit significant differences of APE between the evaluated environments, these environment specific QTL effects were interpreted as Q × E interactions (Figure 3).

The *QLV318RD-19* exhibits positive APE, which was correlated with susceptibility effect or high AUDPC values from the phenotypic evaluation. However, the *QLV681RD-6* and *QAR681D-14* showed negative APEs and correlate well with resistance or low AUDPC values. The *QAR681D-14* is a conditionally neutral QTL, because it was detected only in a specific environment. The QTL *QLV681RD-6* and *QLV318RD-19* are stable, showing different effect levels in dry and rainy seasons. However, the QTL *QLV681RD-6*, detected during rainy and dry seasons, showed a significant APE value only under dry conditions (Figure 3).

### Sequence analysis of chromosome regions containing the identified QTL

The DNA sequences of genome intervals harboring each QTL were analyzed *in silico* for the presence of genes by Blasting in the public databases. The found hits were considered as CBB CDRGs. In total, 29 CDRGs were found and they are distributed in all the regions containing QTL (Supplementary Material 4). The QTL physical intervals comprised 263.3 Kb corresponding to a genetic distance of 8.6 cM with a mean value of 30.6 Kb per 1 cM. On average, one gene each 9.07 Kb was found. However, this ratio varies between the QTL from 0.07 in *QGH681-2.2* region to 0.4 genes per Kb in *QLV681RD-6*. QTL intervals *QGH681-2.2, QLV318RD-19*, and *QGH318-19* which showed the highest interval-lengths (101.1, 80.0, and 58.8 Kb, respectively) contained the highest number of genes (8 genes in each interval). However, the gene density (GD) varied from one gene every 7.3 Kb for *QGH318-19* region to 13.7 Kb for *QGH681-2.2*.

Among the 29 CDRGs co-localizing with the QTL, five genes (17.2%) have not been previously annotated and/or described in the current cassava genome, based on PFAM (Finn et al., 2014), PANTHER (Thomas et al., 2003) and EuKaryotic Orthologous Groups (KOG) (Koonin et al., 2004). The identified candidate genes contain diverse protein domains, without particular domains being most represented. Interestingly, two immunity related genes (IRGs) co-localized with *QLV318RD-19*, but 1.4 Mb apart from each other: one of them coding for a WRKY DNA -binding domain bearing protein and the second coding for a NB-ARC-LRR class of proteins. A gene coding for a subtilisin-like protease was found within the interval of the stable QTL *QLV681RD-6*. Also, a gene containing kinase domain was identified in the *QGH681-2.2* interval (Supplementary Material 4).

## Discussion

In this study, the defense response of 117 genotypes of a F1 mapping population infected with two *Xam* strains was evaluated in different environments: two locations under rainy and dry seasons and under greenhouse controlled conditions, which allowed for the identification of five strain-specific QTL for resistance to CBB. *In silico* analyses of the QTL intervals revealed 29 CDRGs that might operate in the defense response. The strains used in this study are part of a set of *Xam* strains evaluated for virulence in nine cassava accessions. The *Xam318* and *Xam681* showed a high virulent behavior, causing disease in six and eight of nine accessions tested, respectively (Trujillo et al., 2014). Moreover, these strains have been reported as part of prevalent haplotypes described in Colombian *Xam* populations and to be the product of migratory processes between regions in Colombia (Trujillo et al., 2014). The *Xam* populations in these regions remained unexplored since more than one decade (Restrepo, 1999). Although cassava is cultivated in both regions, the grown areas are relatively limited. It remains interesting to examine if the two *Xam* strains used for the resistance test in this study do exist in the *Xam* populations in La Vega, Cundinamarca and Arauca where the studies were performed. This will allow directing the breeding programs toward the resistance to these *Xam* strains for these particular Colombian regions.

The broad sense heritability of cassava plants estimated in this study was 20 and 53% for resistance to *Xam*318 and *Xam*681, respectively. The values are in the range of the previously reported studies of 10–69% (Hahn et al., 1990; Jorge et al., 2000; Fregene et al., 2001; Ly et al., 2013). These values of heritability highlight the important effect of the environmental conditions on plant response to CBB. In particular, the humidity showed a strong influence on the phenotypic response of some of the F1 individuals. Thus, the number of susceptible individuals was higher during the rainy season compared to the dry season in both environments (68 vs. 64 for LV and 60 vs. 44 for Arauca). Several studies have shown a positive (favorable) effect of humidity not only on the speed of symptoms, but also on the growing of *Xam* (Banito et al., 2001; Wydra and Verdier, 2002; Restrepo et al., 2004). Even though the environments with higher humidity seem to favor the disease, temperature also can generate different effects under a particular genotype. This can lead to a change in the plant phenotype, which is defined as phenotypic plasticity (Nicotra et al., 2010). In all the conditions evaluated genotypes that exhibited resistant behavior under some environmental conditions but susceptible in others were found. For example, three genotypes g29, g30, and g116, showed resistance under the dry season but susceptibility under the rainy one, whereas g51 and g97 which were resistant to Xam681 in La Vega during rainy and dry seasons, but susceptible under the rainy season in Arauca. The phenotypic plasticity has been widely described in model and non-model crops for several traits, including resistance to plant pathogens (Agrawal, 1999; Dicke and Hilker, 2003). The individuals showing phenotypic plasticity identified in this study can be used in local breeding programs as superior genotypes adapted to specific environmental conditions as an approach exploiting adaptive plasticity (Nicotra et al., 2010).

Despite that no statistically significantly differences between the transgressive segregants and its parents were found, it is important to consider a clear differential biological behavior of CBB resistance and symptoms for some of these genotypes. In this way and taking into account other factors, these genotypes represent important sources of resistance for future use in cassava breeding programs. In addition, these genotypes can be also considered for future research aiming to better understand the mechanisms of bacterial resistance in cassava. Transgressive segregation of resistance has been previously described in cassava against *Xam* strains (Jorge et al., 2000) as well as for other important traits (Akinwale et al., 2010; Whankaew et al., 2011; Njenga et al., 2014; Thanyasiriwat et al., 2014). This type of heterosis has been explained by the presence of blocks of dominant genes from both parents (Bingham, 1998; Jorge et al., 2000), variations in the chromosome number, chromosome rearrangements (Rieseberg et al., 1999), and even DNA methylation, epigenetics and silencing by small RNAs (Shivaprasad et al., 2012). The highest resistance was scored in the evaluations conducted under controlled conditions. A particular case was the genotype g79, which was categorized as a resistant transgressive phenotype for almost all the conditions evaluated. The genotypes g3, g103, g118, g126, were detected showing extreme resistance in both rainy and dry season against *Xam*318 and *Xam*681 (Supplementary Material 2). These genotypes could be important sources of resistance for future use in cassava breeding programs.

Five strain specific QTL conferring resistance against *Xam* in cassava were identified in this study. Three of the associated QTL were found to be effective against *Xam*318 strain, while two were detected for *Xam*681. Jorge et al. (2000), have reported 12 QTL for resistance to five Colombian *Xam* strains, while Wydra et al. (2004) nine QTL to four *Xam* strains. None of these QTL were identified in the present study, and could be explained by: the lack of common markers between the used genetic maps; by the very different environments where the evaluations were conducted; by the different genetic backgrounds of the mapping populations. The strains used in this study are different from those used in above mentioned reports, and all the QTL we identified were strain-specific. Detection of strain-specific QTL has already been reported for quantitative resistance in several crops such as rice (Li et al., 1999), tomato (Wang et al., 2000; Carmeille et al., 2006), melon (Perchepied et al., 2005), and apple (Calenge et al., 2004). Taken together, these results suggest a strong strain x cultivar x environment interaction. In order to prove this hypothesis, it will be important to consider phenotypic evaluations in multi-environments with the same *Xam* strain as well as to expand the repertoire of strains belonging to the same and different haplotypes.

The use of the APE as a parameter to establish Q x E interaction have been reported in some crops such as wheat (Hao et al., 2011) and in the plant model Arabidopsis (El-Soda et al., 2014). Here, the Q × E interactions were established for all QTL except for those detected under greenhouse conditions. Three QTL showed significant APE values. Remarkably, the QTL detected under dry conditions exhibit a negative APE. This shows once again the important role of the environment and especially of the humidity in favoring CBB disease. The detection of QTL with a significant APE in one environment but not in another, unstable QTL or conditionally neutral QTL, are evidence of the pivotal role of the environment conditions in the instability of the detection of QTL between environments. Conditionally neutral resistance QTL have been reported in some crops like rice (Li et al., 2007), wheat (Ramburan et al., 2004), and apple (Calenge and Durel, 2006). In cassava, in spite of that environmentally unstable QTL for some traits have been reported (Jorge et al., 2001), Q × E interaction for CBB has not been described so far. Although, stable detected QTL are most useful for breeding programs, the mechanisms behind a conditional neutral QTL are useful in plant improvement. These resistance loci indicate the great influence of external conditions, such as environmental factors on the phenotype, thus, they can be exploited in local programs that present particular environmental conditions and adapted pathogens.

All the QTL identified cover in total a physical part of the cassava genome corresponding to 263.3 Kb. In these regions 29 CDRGs are found. With the advent of new high throughput genotyping technologies the number of markers increased exponentially and allowed the construction of genetic high resolutions maps in cassava (Soto et al., 2015). The efforts made recently to sequence the cassava genome allow a better characterization of the QTL intervals, including the number and nature of the genes present in these regions. In spite of associations between candidates genes with QTL have been reported (Faris et al., 1999; Ramalingam et al., 2003), the isolation of genes from QTL regions are scarce. By using a genetic map with higher resolution we were able to identify 29 CDRGs that genetically co-localized with QTL in short interval lengths (2.8 cM in average).

From the 24 annotated genes, two are related to plant immunity and showed to be co-localized with one QTL (*QLV318RD-19)*. Similar findings have been reported for other plant species (Ramalingam et al., 2003; St. Clair, 2010; Lopez, 2011). The resistance to CBB has been classified as a quantitative trait and in cassava Avr-R interactions have not been demonstrated so far. Nevertheless, the presence of genes coding for typical *R* proteins within the QTL intervals supports the finding of an overlapping between qualitative and quantitative resistances.

In the whole repertoire of genes present in the QTL intervals, a kinase co-localized with *QGH681-2.2*. Despite the kinase family being one of the most widely distributed proteins in plant genomes, finding a co-localization of a member of this family with resistance seem not to be by chance. Several proteins of this family have been shown to be involved in plant resistance in model plants (Huard-Chauveau et al., 2013) as well as in some of the most important crops such as barley (Druka et al., 2008), wheat (Fu et al., 2009), and maize (Zuo et al., 2015). The kinases can be an important element in quantitative disease resistance, either as a receptor or as part of the signaling pathway. Kinase proteins have been reported to be involved in defense response pathways in Arabidopsis and in other crops (Roux et al., 2014). In tomato for example, the *Pseudomonas* resistance gene (*Pto*) encodes a Ser/Thr kinase (Oh and Martin, 2011). The large group of kinases has been exploited as “decoys” for the study of plant immunity (van der Hoorn and Kamoun, 2008).

Even though some of the CDRGs present in the QTL regions do not belong to IRGs, they still need to be considered as potential candidate genes for CBB resistance. Conversely, it has been demonstrated that the classical immunity-related proteins could be only a small fraction of the total genes involved in quantitative resistance (Corwin et al., 2016). Cloning of some genes for plant quantitative resistance has given evidence that the corresponding proteins do not belong to any specific group of immunity related proteins or they lack the conserved domains of R proteins. These genes are involved in different functions and processes (Poland et al., 2009; Bryant et al., 2014; Roux et al., 2014). Thus, the gene coding for a subtilisin-like protease that co-localized with the stable and major QTL *QLV681RD-6*, could be considered as an interesting and promising CDRG for several reasons: first, due to fact that it co-localizes with a QTL that explains the highest percentage of phenotypic variance; second, the coding protein subtilisin-like protease has been described to be involved in resistance to pathogens in other crops such as tomato (Tornero et al., 1996; Jordá et al., 1999), grapevine (Figueiredo et al., 2008; Monteiro et al., 2013) and in Arabidopsis (Ramirez et al., 2013; Figueiredo et al., 2014); third, this candidate gene co-localizes with a stable QTL, suggesting that the effect in conferring defense response to *Xam* of this gene is not strongly affected by the environment.

The repertoire of CDRGs co-localizing with the QTL reported here represents a first step in the dissection of the molecular mechanisms that govern CBB resistance in cassava and a new source of genes to be validated through different approaches in the future. With the advent of gene editing methodologies it will be possible to validate the function of these particular genes in CBB resistance (Sander and Joung, 2014). Moreover, this gene repertoire and the associated SNP markers can be used in plant breeding to develop CBB resistant cassava that are adapted to the evaluated regions and other locations with similar environmental conditions.

## Author contributions

JS carried out the CBB phenotyping and QTL analysis. RM conducted part of the CBB phenotyping. JS, AB, and CL wrote the manuscript and contributed to the interpretation of the results. BM and JL performed the statistical analysis. JS and CL designed the experiments. CL coordinated the project. FG performed the CDRGs analysis. All authors read and approved the final manuscript.

### Conflict of interest statement

The authors declare that the research was conducted in the absence of any commercial or financial relationships that could be construed as a potential conflict of interest.

## References

[B1] Agrawal A. A. (1999). Induced plant defense: evolution of induction and adaptive phenotypic plasticity, in Inducible Plant Defenses Against Pathogens and Herbivores: Biochemistry, Ecology, and Agriculture, eds Agrawal A. A. Tuzun S. Bent L. (St. Paul, MN: American Phytopathological Society Press), 251–268.

[B2] Akinwale M. G. Aladesanwa R. D. Akinyele B. O. Dixon A. G. O. Odiyi A. C. (2010). Inheritance of B-carotene in cassava (*Manihot esculenta* crantz). Int. J. Genet. Mol. Biol. 2, 198–201.

[B3] Anderson J. T. Lee C. R. Rushworth C. A. Colautti R. I. Mitchell-Olds T. (2013). Genetic trade-offs and conditional neutrality contribute to local adaptation. Mol. Ecol. 22, 699–708. 10.1111/j.1365-294X.2012.05522.x 22420446 PMC3492549

[B4] Anderson J. Wagner M. Rushworth C. Prasad K. Mitchell-Olds T. (2014). The evolution of quantitative traits in complex environments. Heredity 11233, 4–12. 10.1038/hdy.2013.33 23612691 PMC3860162

[B5] Banito A. Kpémoua K. E. Wydra K. Rudolph K. (2001). Bacterial blight of cassava in Togo: its importance, the virulence of the pathogen and the resistance of varieties, in Plant Pathogenic Bacteria (Springer), 259–264.

[B6] Bart R. Cohn M. Kassen A. McCallum E. J. Shybut M. Petriello A.. (2012). PNAS Plus: high-throughput genomic sequencing of cassava bacterial blight strains identifies conserved effectors to target for durable resistance. Proc. Natl. Acad. Sci. U.S.A. 109, E1972–E1979. 10.1073/pnas.1208003109 22699502 PMC3396514

[B7] Bates D. Maechler M. Bolker B. Walker S. (2015). lme4: Linear Mixed-Effects Models Using Eigen and S4. R package version 1.1-7. 2014. Institute for Statistics and Mathematics of WU website Availabe online at: http://CRAN.R-project.org/package=lme4. Accessed March 18.

[B8] Bingham E. T. (1998). Role of chromosome blocks in heterosis and estimates of dominance and overdominance, in Concepts and Breeding of Heterosis in Crop Plants, eds Larnkey K. R. Staub J. E. (Madison, WI: Crop Science Society of America), 71–87.

[B9] Broman K. W. (2015). R/qtlcharts: interactive graphics for quantitative trait locus mapping. Genetics 199, 359–361. 10.1534/genetics.114.172742 25527287 PMC4317647

[B10] Bryant R. R. M. McGrann G. R. D. Mitchell A. R. Schoonbeek H. Boyd L. A. Uauy C.. (2014). A change in temperature modulates defence to yellow (stripe) rust in wheat line UC1041 independently of resistance gene Yr36. BMC Plant Biol. 14:10. 10.1186/1471-2229-14-10 24397376 PMC3898064

[B11] Calenge F. Durel C. E. (2006). Both stable and unstable QTLs for resistance to powdery mildew are detected in apple after four years of field assessments. Mol. Breed. 17, 329–339. 10.1007/s11032-006-9004-7

[B12] Calenge F. Faure A. Goerre M. Gebhardt C. de Weg W. E. Parisi L.. (2004). Quantitative trait loci (QTL) analysis reveals both broad-spectrum and isolate-specific QTL for scab resistance in an apple progeny challenged with eight isolates of Venturia inaequalis. Phytopathology 94, 370–379. 10.1094/PHYTO.2004.94.4.370 18944113

[B13] Carmeille A. Caranta C. Dintinger J. Prior P. Luisetti J. Besse P. (2006). Identification of QTLs for Ralstonia solanacearum race 3-phylotype II resistance in tomato. Theor. Appl. Genet. 113, 110–121. 10.1007/s00122-006-0277-3 16614830

[B14] Ceballos H. Okogbenin E. Pérez J. C. López-Valle L. A. B. Debouck D. (2010). Cassava, in Root and Tuber Crops, ed Bradshaw J. E. (New York, NY: Springer), 53–96.

[B15] Corwin J. A. Copeland D. Feusier J. Subedy A. Eshbaugh R. Palmer C.. (2016). The quantitative basis of the arabidopsis innate immune system to endemic pathogens depends on pathogen genetics. PLoS Genetics. 12:e1005789. 10.1371/journal.pgen.1005789 26866607 PMC4750985

[B16] Dicke M. Hilker M. (2003). Induced plant defences: from molecular biology to evolutionary ecology. Basic Appl. Ecol. 4, 3–14. 10.1078/1439-1791-00129

[B17] Druka A. Potokina E. Luo Z. Bonar N. Druka I. Zhang L.. (2008). Exploiting regulatory variation to identify genes underlying quantitative resistance to the wheat stem rust pathogen *Puccinia graminis* f. sp. tritici in barley. Theor. Appl. Genet. 117, 261–272. 10.1007/s00122-008-0771-x 18542913

[B18] El-Soda M. Malosetti M. Zwaan B. J. Koornneef M. Aarts M. G. M. (2014). Genotype x environment interaction QTL mapping in plants: lessons from Arabidopsis. Trends Plant Sci. 19, 390–398. 10.1016/j.tplants.2014.01.001 24491827

[B19] Faris J. D. Li W. L. Liu D. J. Chen P. D. Gill B. S. (1999). Candidate gene analysis of quantitative disease resistance in wheat. Theor. Appl. Genet. 98, 219–225. 10.1007/s001220051061

[B20] Figueiredo A. Fortes A. M. Ferreira S. Sebastiana M. Choi Y. H. Sousa L.. (2008). Transcriptional and metabolic profiling of grape (*Vitis vinifera* L.) leaves unravel possible innate resistance against pathogenic fungi. J. Exp. Bot. 59, 3371–3381. 10.1093/jxb/ern187 18648103

[B21] Figueiredo A. Monteiro F. Sebastiana M. (2014). Subtilisin-like proteases in plant–pathogen recognition and immune priming: a perspective. Front. Plant Sci. 5:739. 10.3389/fpls.2014.00739 25566306 PMC4271589

[B22] Finn R. D. Bateman A. Clements J. Coggill P. Eberhardt R. Y. Eddy S. R.. (2014). Pfam: The protein families database. Nucl. Acids Res. 42, D222–D230. 10.1093/nar/gkt1223 24288371 PMC3965110

[B23] Fregene M. Angel F. Gómez R. Rodr'iguez F. Chavarriaga P. Roca W. (1997). A molecular genetic map of cassava (*Manihot esculenta* Crantz). Theor. Appl. Genet. 95, 431–441.

[B24] Fregene M. Okogbenin E. Mba C. Angel F. Suarez M. C. Janneth G. (2001). Genome mapping in cassava improvement: challenges, achievements and opportunities. Euphytica 120, 159–165. 10.1023/A:1017565317940

[B25] Frutos-Bernal E. Galindo M. P. (2012). GGEBiplotGUI: Interactive GGE Biplots in R [Programa informático]. Salamanca, España: Departamento de Estadística, Universidad de Salamanca Available online at: http://CRAN.r-project.org/web/packages/GGEBiplotGUI/index.html

[B26] Fu D. Uauy C. Distelfeld A. Blechl A. Epstein L. Chen X.. (2009). A kinase-START gene confers temperature-dependent resistance to wheat stripe rust. Science 323, 1357–1360. 10.1126/science.1166289 19228999 PMC4737487

[B27] Fukuoka S. Saka N. Koga H. Ono K. Shimizu T. Ebana K.. (2009). Loss of function of a proline-containing protein confers durable disease resistance in rice. Science 325, 998–1001. 10.1126/science.1175550 19696351

[B28] Gebhardt C. Valkonen J. P. T. (2001). Organization of genes controlling disease resistance in the potato genome. Annu. Rev. Phytopathol. 39, 79–102. 10.1146/annurev.phyto.39.1.79 11701860

[B29] Guimaraes R. L. Stotz H. U. (2004). Oxalate production by Sclerotinia sclerotiorum deregulates guard cells during infection. Plant Physiol. 136, 3703–3711. 10.1104/pp.104.049650 15502012 PMC527168

[B30] Hahn S. K. Bai K. V. Asiedu R. (1990). Tetraploids, triploids, and 2n pollen from diploid interspecific crosses with cassava. Theor. Appl. Genet. 79, 433–439. 10.1007/BF00226148 24226443

[B31] Hahn S. K. Howland A. K. Okoli C. A. (1974). Breeding for resistance to cassava bacterial blight at IITA, in Workshop on Cassava Bacterial Blight in Nigeria, eds Okpala E. U. Glaser H. J. (Umudikey).

[B32] Hahn S. K. Howland A. K. Terry E. R. (1980). Correlated resistance of cassava to mosaic and bacterial blight diseases. Euphytica 29, 305–311. 10.1007/BF00025127

[B33] Hao Y. Chen Z. Wang Y. Bland D. Buck J. Brown-Guedira G.. (2011). Characterization of a major QTL for adult plant resistance to stripe rust in US soft red winter wheat. Theoret. Appl. Genet. 123, 1401–1411. 10.1007/s00122-011-1675-8 21830107

[B34] Holland J. B. (2006). Estimating genotypic correlations and their standard errors using multivariate restricted maximum likelihood estimation with SAS Proc MIXED. Crop Sci. 46, 642–654. 10.2135/cropsci2005.0191

[B35] Howeler R. L. Thomas N. Holst Sanjuán K. Sanjuán K. H. Quirós H. Isebrands J. G. (2013). Save and Grow: Cassava. A guide to Sustainable Production Intensification Produire plus Avec Moins Ahorrar Para crecer. Roma: FAO.

[B36] Huard-Chauveau C. Perchepied L. Debieu M. Rivas S. Kroj T. Kars I.. (2013). An atypical kinase under balancing selection confers broad-spectrum disease resistance in Arabidopsis. PLoS Genet. 9:e1003766. 10.1371/journal.pgen.1003766 24068949 PMC3772041

[B37] Jeger M. J. Viljanen-Rollinson S. L. H. (2001). The use of the area under the disease-progress curve (AUDPC) to assess quantitative disease resistance in crop cultivars. Theor. Appl. Genet. 102, 32–40. 10.1007/s001220051615

[B38] Jinks J. L. Jones R. M. (1958). Estimation of the components of heterosis. Genetics 43, 223. 17247752 10.1093/genetics/43.2.223 PMC1209876

[B39] Jordá L. Coego A. Conejero V. Vera P. (1999). A genomic cluster containing four differentially regulated subtilisin-like processing protease genes is in tomato plants. J. Biol. Chem. 274, 2360–2365. 10.1074/jbc.274.4.2360 9891003

[B40] Jorge V. Fregene M. A. Duque M. C. Bonierbale M. W. Tohme J. Verdier V. (2000). Genetic mapping of resistance to bacterial blight disease in cassava (*Manihot esculenta* Crantz). Theor. Appl. Genet. 101, 865–872. 10.1007/s001220051554

[B41] Jorge V. Fregene M. Vélez C. M. Duque M. C. Tohme J. Verdier V. (2001). QTL analysis of field resistance to *Xanthomonas axonopodis* pv. manihotis in cassava. Theor. Appl. Genet. 102, 564–571. 10.1007/s001220051683

[B42] Jorgensen T. H. (2012). The effect of environmental heterogeneity on RPW8-mediated resistance to powdery mildews in *Arabidopsis thaliana*. Ann. Bot. 109, 833–842. 10.1093/aob/mcr320 22234559 PMC3286285

[B43] Koonin E. V. Fedorova N. D. Jackson J. D. Jacobs A. R. Krylov D. M. Makarova K. S.. (2004). A comprehensive evolutionary classification of proteins encoded in complete eukaryotic genomes. Genome Biol. 5:R7. 10.1186/gb-2004-5-2-r7 14759257 PMC395751

[B44] Kou Y. Wang S. (2010). Broad-spectrum and durability: understanding of quantitative disease resistance. Curr. Opin. Plant Biol. 13, 181–185. 10.1016/j.pbi.2009.12.010 20097118

[B45] Kpémoua K. Boher B. Nicole M. Calatayud P. Geiger J.-P. (1996). Cytochemistry of defense responses in cassava infected by *Xanthomonas campestris* pv. manihotis. Can. J. Microbiol. 42, 1131–1143. 10.1139/m96-145

[B46] Li Y. B. Wu C. J. Jiang G. H. Wang L. Q. He Y. Q. (2007). Dynamic analyses of rice blast resistance for the assessment of genetic and environmental effects. Plant Breed. 126, 541–547. 10.1111/j.1439-0523.2007.01409.x

[B47] Li Z.-K. Luo L. J. Mei H. W. Paterson A. H. Zhao X. H. Zhong D. B.. (1999). A “defeated” rice resistance gene acts as a QTL against a virulent strain of *Xanthomonas oryzae* pv. oryzae. Mol. Gen. Genet. 261, 58–63. 10.1007/s004380050941 10071210

[B48] Liu X. Huang B. Lin J. Fei J. Chen Z. Pang Y.. (2006). A novel pathogenesis-related protein (SsPR10) from Solanum surattense with ribonucleolytic and antimicrobial activity is stress-and pathogen-inducible. J. Plant Physiol. 163, 546–556. 10.1016/j.jplph.2005.04.031 16473659

[B49] Lokko Y. Gedil M. Dixon A. (2004). QTLs associated with resistance to the cassava mosaic disease, in Proceedings of the 4th International Crop Science Congress (Brisbane, QLD).

[B50] Lopez C. (2011). Descifrando las bases moleculares de la resistencia cuantitativa. Acta Biol. Colomb. 16, 3.

[B51] López C. E. Bernal A. J. (2012). Cassava bacterial blight: using genomics for the elucidation and management of an old problem. Trop. Plant Biol. 5, 117–126. 10.1007/s12042-011-9092-3

[B52] Lopez C. E. Quesada-Ocampo L. M. Bohorquez A. Duque M. C. Vargas J. Tohme J.. (2007). Mapping EST-derived SSRs and ESTs involved in resistance to bacterial blight in *Manihot esculenta*. Genome 50, 1078–1088. 10.1139/G07-087 18059536

[B53] Lopez C. E. Zuluaga A. P. Cooke R. Delseny M. Tohme J. Verdier V. (2003). Isolation of resistance gene candidates (RGCs) and characterization of an RGC cluster in cassava. Mol. Genet. Genom. 269, 658–671. 10.1007/s00438-003-0868-5 12827500

[B54] Lozano J. (1986). Cassava bacterial blight: a manageable disease. Plant Dis. 70, 1089–1093. 10.1094/PD-70-1089

[B55] Lozano R. Hamblin M. T. Prochnik S. Jannink J.-L. (2015). Identification and distribution of the NBS-LRR gene family in the Cassava genome. BMC Genomics 16:9. 10.1186/s12864-015-1554-9 25948536 PMC4422547

[B56] Ly D. Hamblin M. Rabbi I. Melaku G. Bakare M. Gauch H. G. (2013). Relatedness and genotype x environment interaction affect prediction accuracies in genomic selection: a study in cassava. Crop Sci. 53, 1312–1325. 10.2135/cropsci2012.11.0653

[B57] Mansfield J. Genin S. Magori S. Citovsky V. Sriariyanum M. Ronald P.. (2012). Top 10 plant pathogenic bacteria in molecular plant pathology. Mol. Plant Pathol. 13, 614–629. 10.1111/j.1364-3703.2012.00804.x 22672649 PMC6638704

[B58] Mba R. E. C. Stephenson P. Edwards K. Melzer S. Nkumbira J. Gullberg U. (2001). Simple sequence repeat (SSR) markers survey of the cassava (*Manihot esculenta* Crantz) genome: towards an SSR-based molecular genetic map of cassava. Theor. Appl. Genet. 102, 21–31. 10.1007/s001220051614

[B59] Meng L. Li H. Zhang L. Wang J. (2015). QTL IciMapping: integrated software for genetic linkage map construction and quantitative trait locus mapping in biparental populations. Crop. J. 3, 269–283. 10.1016/j.cj.2015.01.001

[B60] Mitchell-Olds T. (2013). Selection on QTL and complex traits in complex environments. Mol. Ecol. 22, 3427–3429. 10.1111/mec.12345 23967451 PMC3884901

[B61] Monteiro F. Sebastiana M. Pais M. S. Figueiredo A. (2013). Reference gene selection and validation for the early responses to downy mildew infection in susceptible and resistant Vitis vinifera cultivars. PLoS ONE 8:e72998. 10.1371/journal.pone.0072998 24023800 PMC3762845

[B62] Nicotra A. B. Atkin O. K. Bonser S. P. Davidson A. M. Finnegan E. J. Mathesius U.. (2010). Plant phenotypic plasticity in a changing climate. Trends Plant Sci. 15, 684–692. 10.1016/j.tplants.2010.09.008 20970368

[B63] Njenga P. Edema R. Kamau J. (2014). Combining ability for beta-carotene and important quantitative traits in a cassava F1 population. J. Plant Breed. Crop Sci. 6, 24–30. 10.5897/JPBCS12.069

[B64] Oh C.-S. Martin G. B. (2011). Effector-triggered immunity mediated by the Pto kinase. Trends Plant Sci. 16, 132–140. 10.1016/j.tplants.2010.11.001 21112235

[B65] Perchepied L. Dogimont C. Pitrat M. (2005). Strain-specific and recessive QTLs involved in the control of partial resistance to *Fusarium oxysporum* f. sp. melonis race 1.2 in a recombinant inbred line population of melon. Theor. Appl. Genet. 111, 65–74. 10.1007/s00122-005-1991-y 15834544

[B66] Poland J. A. Balint-Kurti P. J. Wisser R. J. Pratt R. C. Nelson R. J. (2009). Shades of gray: the world of quantitative disease resistance. Trends Plant Sci. 14, 21–29. 10.1016/j.tplants.2008.10.006 19062327

[B67] Rabbi I. Y. Hamblin M. T. Kumar P. L. Gedil M. A. Ikpan A. S. Jannink J.-L.. (2014). High-resolution mapping of resistance to cassava mosaic geminiviruses in cassava using genotyping-by-sequencing and its implications for breeding. Virus Res. 186, 87–96. 10.1016/j.virusres.2013.12.028 24389096

[B68] Ramalingam J. Cruz C. M. V. Kukreja K. Chittoor J. M. Wu J.-L. Lee S. W.. (2003). Candidate defense genes from rice, barley, and maize and their association with qualitative and quantitative resistance in rice. Mol. Plant Microbe Interact. 16, 14–24. 10.1094/MPMI.2003.16.1.14 12580278

[B69] Ramburan V. P. Pretorius Z. A. Louw J. H. Boyd L. A. Smith P. H. Boshoff W. H. P.. (2004). A genetic analysis of adult plant resistance to stripe rust in the wheat cultivar Kariega. Theor. Appl. Genet. 108, 1426–1433. 10.1007/s00122-003-1567-7 14963651

[B70] Ramirez V. López A. Mauch-Mani B. Gil M. J. Vera P. (2013). An extracellular subtilase switch for immune priming in Arabidopsis. PLoS Pathog. 9:e1003445. 10.1371/journal.ppat.1003445 23818851 PMC3688555

[B71] Restrepo S. (1999). Etude de la Structure des Populations de Xanthomonas axonopodis pv. Manihotis en Colombie. Thèse Nouveau Doctorat Notes, Travaux Universitaires, Travaux.

[B72] Restrepo S. Duque M. C. Verdier V. (2000). Characterization of pathotypes among isolates of *Xanthomonas axonopodis* pv. manihotis in Colombia. Plant Pathol. 49, 680–687. 10.1046/j.1365-3059.2000.00513.x

[B73] Restrepo S. Velez C. M. Duque M. C. Verdier V. (2004). Genetic structure and population dynamics of *Xanthomonas axonopodis* pv. manihotis in Colombia from 1995 to 1999. Appl. Environ. Microbiol. 70, 255–261. 10.1128/AEM.70.1.255-261.2004 14711649 PMC321237

[B74] Rieseberg L. H. Archer M. A. Wayne R. K. (1999). Transgressive segregation, adaptation and speciation. Heredity 83:372. 10.1038/sj.hdy.6886170 10583537

[B75] Ripley B. D. (2001). The R project in statistical computing. MSOR Connect. Newsl. LTSN Maths Stats. OR Netw. 1, 23–25. 10.11120/msor.2001.01010023

[B76] Roux F. Voisin D. Badet T. Balagé C. Barlet X. Huard-Chauveau C.. (2014). Resistance to phytopathogense tutti quanti: placing plant quantitative disease resistance on the map. Mol. Plant Pathol. 15, 427–432. 10.1111/mpp.12138 24796392 PMC6638617

[B77] Sander J. D. Joung J. K. (2014). CRISPR-Cas systems for genome editing, regulation and targeting. Nat. Biotechnol. 32, 347–355. 10.1038/nbt.2842 24584096 PMC4022601

[B78] Sandino T. López-Kleine L. López C. Marquinez X. (2015). Characterization of the morphological response of susceptible and resistant varieties of cassava (*Manihot esculenta* Crantz) to vascular bacterial blight caused by *Xanthomonas axonopodis* pv manihotis. Summa Phytopathol. 41, 94–100. 10.1590/0100-5405/2031

[B79] Shaner G. Finney R. E. (1977). The effect of nitrogen fertilization on the expression of slow-mildewing resistance in Knox wheat. Phytopathology 67, 1051–1056. 10.1094/Phyto-67-1051

[B80] Shivaprasad P. V. Dunn R. M. Santos B. A. C. M. Bassett A. Baulcombe D. C. (2012). Extraordinary transgressive phenotypes of hybrid tomato are influenced by epigenetics and small silencing RNAs. EMBO J. 31, 257–266. 10.1038/emboj.2011.458 22179699 PMC3261569

[B81] Soto J. C. Ortiz J. F. Perlaza-Jiménez L. Vásquez A. X. Lopez-Lavalle L. A. B. Mathew B.. (2015). A genetic map of cassava (*Manihot esculenta* Crantz) with integrated physical mapping of immunity-related genes. BMC Genomics 16:190. 10.1186/s12864-015-1397-4 25887443 PMC4417308

[B82] St. Clair D. A. (2010). Quantitative disease resistance and quantitative resistance loci in breeding. Annu. Rev. Phytopathol. 48, 247–268. 10.1146/annurev-phyto-080508-081904 19400646

[B83] Taylor N. J. Fauquet C. M. Tohme J. (2012). Overview of cassava special issue. Trop. Plant Biol. 5, 1–3. 10.1007/s12042-012-9098-5 PMC3322327 22523606

[B84] Thanyasiriwat T. Sraphet S. Whankaew S. Boonseng O. Bao J. Lightfoot D. A.. (2014). Quantitative trait loci and candidate genes associated with starch pasting viscosity characteristics in cassava (*Manihot esculenta* Crantz). Plant Biol. 16, 197–207. 10.1111/plb.12022 23614826

[B85] Thomas P. D. Campbell M. J. Kejariwal A. Mi H. Karlak B. (2003). PANTHER: a library of protein families and subfamilies indexed by function. Genome Res. 13, 2129–2141. 10.1101/gr.772403 12952881 PMC403709

[B86] Tornero P. Conejero V. Vera P. (1996). Primary structure and expression of a pathogen-induced protease (PR-P69) in tomato plants: similarity of functional domains to subtilisin-like endoproteases. Proc. Natil. Acad. Sci. U.S.A. 93, 6332–6337. 10.1073/pnas.93.13.6332 8692815 PMC39022

[B87] Trujillo C. A. Ochoa J. C. Mideros M. F. Restrepo S. López C. Bernal A. (2014). A complex population structure of the Cassava Pathogen *Xanthomonas axonopodis* pv. manihotis in recent years in the Caribbean Region of Colombia. Microb. Ecol. 68, 155–167. 10.1007/s00248-014-0411-8 24760168

[B88] van der Hoorn R. A. L. Kamoun S. (2008). From guard to decoy: a new model for perception of plant pathogen effectors. Plant Cell 20, 2009–2017. 10.1105/tpc.108.060194 18723576 PMC2553620

[B89] Van Loon L. C. Rep M. Pieterse C. M. J. (2006). Significance of inducible defense-related proteins in infected plants. Annu. Rev. Phytopathol. 44, 135–162. 10.1146/annurev.phyto.44.070505.143425 16602946

[B90] Verdier V. Boher B. Maraite H. Geiger J.-P. (1994). Pathological and molecular characterization of Xanthomonas campestris strains causing diseases of cassava (*Manihot esculenta*). Appl. Environ. Microbiol. 60, 4478–4486. 16349463 10.1128/aem.60.12.4478-4486.1994 PMC202008

[B91] Voorrips R. E. (2002). MapChart: software for the graphical presentation of linkage maps and QTLs. J. Hered. 93, 77–78. 10.1093/jhered/93.1.77 12011185

[B92] Wang J.-F. Olivier J. Thoquet P. Mangin B. Sauviac L. Grimsley N. H. (2000). Resistance of tomato line Hawaii7996 to Ralstonia solanacearum Pss4 in Taiwan is controlled mainly by a major strain-specific locus. Mol. Plant Microbe Interact. 13, 6–13. 10.1094/MPMI.2000.13.1.6 10656580

[B93] Weinig C. Schmitt J. (2004). Environmental effects on the expression of quantitative trait loci and implications for phenotypic evolution. Bioscience 54, 627–635. 10.1641/0006-3568(2004)054[0627:EEOTEO]2.0.CO;2

[B94] Whankaew S. Poopear S. Kanjanawattanawong S. Tangphatsornruang S. Boonseng O. Lightfoot D. A.. (2011). A genome scan for quantitative trait loci affecting cyanogenic potential of cassava root in an outbred population. BMC Genomics 12:266. 10.1186/1471-2164-12-266 21609492 PMC3123654

[B95] Wonni I. Ouedraogo L. Dao S. Tekete C. Koita O. Taghouti G. (2015). First report of Cassava Bacterial Blight caused by *Xanthomonas axonopodis* pv. manihotis in Burkina Faso. Plant Disease. 99:551 10.1094/PDIS-03-14-0302-PDN

[B96] Wydra K. Verdier V. (2002). Occurrence of cassava diseases in relation to environmental, agronomic and plant characteristics. Agric. Ecosyst. Environ. 93, 211–226. 10.1016/S0167-8809(01)00349-8

[B97] Wydra K. Zinsou V. Jorge V. Verdier V. (2004). Identification of pathotypes of *Xanthomonas axonopodis* pv. manihotis in Africa anddetection of quantitative trait loci and markers for resistance to bacterial blight of cassava. Phytopathology 94, 1084–1093. 10.1094/PHYTO.2004.94.10.1084 18943797

[B98] Yan W. Hunt L. A. Sheng Q. Szlavnics Z. (2000). Cultivar evaluation and mega-environment investigation based on the GGE biplot. Crop Sci. 40, 597–605. 10.2135/cropsci2000.403597x

[B99] Yan W. Tinker N. A. (2006). Biplot analysis of multi-environment trial data: principles and applications. Can. J. Plant Sci. 86, 623–645. 10.4141/P05-169

[B100] Zuo W. Chao Q. Zhang N. Ye J. Tan G. Li B.. (2015). A maize wall-associated kinase confers quantitative resistance to head smut. Nat. Genet. 47, 151–157. 10.1038/ng.3170 25531751

