# Supplementary material for: Major Novel QTL for Resistance to Cassava Bacterial Blight Identified through a Multi-Environmental Analysis
Source: Front Plant Sci. 2017 Jul 5;8:1169. doi: 10.3389/fpls.2017.01169 (PMC5496946; doi:10.3389/fpls.2017.01169)
Supplement: Supplementary file 1 [file Table1.PDF]

Major novel QTL for resistance to cassava bacterial blight identified through a multi-environmental analysis

Johana Carolina Soto Sedano[1] jcsotos@unal.edu.co, Rubén Eduardo Mora Moreno[1] remoram@unal.edu.co, Boby Mathew[2] boby.mathew@hotmail.com, Jens Léon[2] ulp201@uni-bonn.de, Fabio Andrés Gómez Cano[1-3] gomezcano.1@osu.edu[1-3] Agim Ballvora[2] ballvora@uni-bonn.de, Camilo Ernesto López Carrascal[1*] celopezc@unal.edu.co, telephone number 571- 3165000 ext.11328 *Corresponding autor

[1] Manihot Biotec Laboratory, Biology department, Universidad Nacional de Colombia, Bogotá, Colombia.

[2] INRES-Plant Breeding University of Bonn, Bonn, Germany.

[3 current address] Center for Applied Plant Sciences (CAPS), The Ohio State University, Columbus, USA.

Online Resource 2 Phenotypic responses to Xam318 and Xam681 during multi-environment evaluation. K= genotype, R= resistant, S= susceptible. (year (2013= rainy season, 2014= dry season; Location (ara= Arauca, lv= La Vega, gh= Greenhouse; Xam strain (318 or 681).

	2013ar318		2014ar318		2013lv318		2014lv318		2013gh318		2014gh318	
g1	1,36	R	1,29	R	1,45	R	1,29	R	1,48	R	1,5	R
g100	1,39	R	1,55	R	1,75	S	1,75	S	1,28	R	1,28	R
g101	1,36	R	1,43	R	1,55	R	1,44	R	1,49	R	1,5	R
g102	1,30	R	1,48	R	1,67	S	1,67	S	1,35	R	1,34	R
g103	1,30	R	1,42	R	1,53	R	1,54	R	1,3	R	1,3	R
g108	1,50	R	1,49	R	1,69	S	1,61	S	1,27	R	1,27	R
g109	1,47	R	1,42	R	1,48	R	1,48	R	1,67	S	1,66	S
g11	1,57	R	1,20	R	1,36	R	1,15	R	1,24	R	1,25	R
g111	1,33	R	1,34	R	1,55	R	1,46	R	1,59	R	1,59	R
g116	1,77	S	1,58	R	1,3	R	1,48	R	1,18	R	1,2	R
g118	1,33	R	1,36	R	1,27	R	1,31	R	1,34	R	1,3	R
g121	1,76	S	1,76	S	1,31	R	1,31	R	1,29	R	1,31	R
g124	1,55	R	1,70	S	1,78	S	1,78	S	1,77	S	1,77	S
g125	1,55	R	1,53	R	1,5	R	1,44	R	1,35	R	1,36	R
g126	1,49	R	1,46	R	1,56	R	1,54	R	1,47	R	1,47	R
g128	1,48	R	1,50	R	1,25	R	1,29	R	1,62	S	1,63	S
g139	1,43	R	1,46	R	1,46	R	1,48	R	1,8	S	1,8	S
g14	1,58	R	1,31	R	1,81	S	1,45	R	1,43	R	1,43	R
g143	1,46	R	1,38	R	1,16	R	1,2	R	1,72	S	1,72	S
g145	1,35	R	1,34	R	1,63	S	1,61	S	1,77	S	1,77	S
g146	1,44	R	1,45	R	1,23	R	1,26	R	1,77	S	1,67	S
g147	1,36	R	1,32	R	1,77	S	1,79	S	1,46	R	1,46	R
g15	1,56	R	1,23	R	1,41	R	1,24	R	1,72	S	1,72	S
g18	1,48	R	1,36	R	1,52	R	1,45	R	1,64	S	1,66	S
g2	1,39	R	1,15	R	1,36	R	1,14	R	1,32	R	1,33	R
g21	1,42	R	1,28	R	1,49	R	1,34	R	1,39	R	1,39	R
g23	1,65	S	1,65	S	1,79	S	1,79	S	1,47	R	1,49	R
g24	1,27	R	1,27	R	1,42	R	1,42	R	1,28	R	1,29	R
g25	1,63	S	1,63	S	1,45	R	1,17	R	1,29	R	1,29	R
g26	1,46	R	1,46	R	1,71	S	1,71	S	1,37	R	1,37	R
g29	1,65	S	1,49	R	1,49	R	1,65	S	1,87	S	1,85	S
g3	1,34	R	0,97	R	1,54	R	1,2	R	1,55	R	1,55	R
g30	1,62	S	1,54	R	1,34	R	1,34	R	1,63	S	1,62	S
g31	1,35	R	1,38	R	1,5	R	1,48	R	1,65	S	1,66	S
g33	1,38	R	1,38	R	1,34	R	1,34	R	1,15	R	1,17	R
g4	1,38	R	1,29	R	1,32	R	1,04	R	1,51	R	1,51	R
g40	1,67	S	1,48	R	1,36	R	1,36	R	1,47	R	1,48	R
g41	1,69	S	1,69	S	1,69	S	1,69	S	1,75	S	1,75	S
g42	1,51	R	1,61	S	1,72	S	1,72	S	1,4	R	1,4	R
g45	1,52	R	1,63	S	1,68	S	1,66	S	1,61	S	1,61	S
g46	1,41	R	1,69	S	1,82	S	1,82	S	1,29	R	1,28	R
g47	1,65	S	1,43	R	1,38	R	1,38	R	1,23	R	1,22	R
g5	1,61	S	1,15	R	1,38	R	1,1	R	1,48	R	1,48	R
g51	1,71	S	1,71	S	1,35	R	1,35	R	1,64	S	1,64	S
g52	1,56	R	1,46	R	1,32	R	1,34	R	1,47	R	1,46	R
g53	1,61	S	1,59	R	1,46	R	1,43	R	1,49	R	1,49	R
g55	1,73	S	1,71	S	1,49	R	1,47	R	1,45	R	1,49	R
g56	1,55	R	1,54	R	1,46	R	1,44	R	1,76	S	1,78	S
g57	1,60	S	1,63	S	1,34	R	1,37	R	1,72	S	1,72	S
g6	1,48	R	1,35	R	1,45	R	1,13	R	1,49	R	1,49	R
g62	1,56	R	1,58	R	1,41	R	1,43	R	1,71	S	1,71	S
g63	1,33	R	1,35	R	1,66	S	1,67	S	1,62	S	1,62	S
g64	1,55	R	1,58	R	1,46	R	1,49	R	1,75	S	1,79	S
g70	1,54	R	1,47	R	1,72	S	1,75	S	1,51	R	1,51	R
g74	1,52	R	1,52	R	1,75	S	1,75	S	1,51	R	1,49	R
g75	1,78	S	1,78	S	1,47	R	1,47	R	1,51	R	1,51	R
g77	1,43	R	1,38	R	1,37	R	1,42	R	1,2	R	1,2	R
g78	1,56	R	1,46	R	1,47	R	1,48	R	1,68	S	1,59	R
g79	1,24	R	1,24	R	1,62	S	1,62	S	1,37	R	1,38	R
g80	1,27	R	1,27	R	1,17	R	1,17	R	1,49	R	1,49	R
g81	1,40	R	1,40	R	1,69	S	1,69	S	1,15	R	1,13	R
g82	1,32	R	1,32	R	1,61	S	1,61	S	1,57	R	1,55	R
g85	1,33	R	1,40	R	1,55	R	1,62	S	1,24	R	1,24	R
g88	1,49	R	1,49	R	1,35	R	1,35	R	1,39	R	1,39	R
g91	1,68	S	1,68	S	1,55	R	1,55	R	1,74	S	1,74	S
g92	1,64	S	1,64	S	1,35	R	1,35	R	1,71	S	1,69	S
g95	1,48	R	1,48	R	1,54	R	1,54	R	1,59	R	1,53	R
g96	1,67	S	1,67	S	1,32	R	1,32	R	1,58	R	1,56	R
g97	1,37	R	1,33	R	1,63	S	1,6	S	1,57	R	1,59	R
g99	1,49	R	1,47	R	1,44	R	1,46	R	1,79	S	1,8	S

	2013ar681		2014ar681		2013lv681		2014lv681		2013gh681		2014gh681	
g1	1,41	R	1,55	R	1,64	S	1,58	R	1,73	S	1,71	S
g10	1,62	S	1,4	R	1,33	R	1,33	R	1,64	S	1,6	S
g100	1,45	R	1,47	R	1,42	R	1,37	R	1,2	R	1,18	R
g101	1,68	S	1,68	S	1,43	R	1,41	R	1,32	R	1,32	R
g102	1,54	R	1,52	R	1,46	R	1,46	R	1,54	R	1,53	R
g103	1,46	R	1,46	R	1,46	R	1,4	R	1,42	R	1,41	R
g108	1,5	R	1,5	R	1,47	R	1,49	R	1,29	R	1,29	R
g109	1,81	S	1,81	S	1,9	S	1,9	S	1,66	S	1,65	S
g11	1,8	S	1,16	R	1,81	S	1,39	R	1,62	S	1,61	S
g111	1,54	R	1,5	R	1,49	R	1,46	R	1,65	S	1,65	S
g116	1,5	R	1,46	R	1,54	R	1,54	R	1,15	R	1,14	R
g118	1,52	R	1,51	R	1,57	R	1,55	R	1,51	R	1,5	R
g121	1,47	R	1,37	R	1,48	R	1,29	R	1,18	R	1,17	R
g124	1,66	S	1,66	S	1,68	S	1,68	S	1,4	R	1,39	R
g125	1,54	R	1,55	R	1,68	S	1,73	S	1,39	R	1,39	R
g126	1,38	R	1,41	R	1,55	R	1,56	R	1,39	R	1,38	R
g128	1,44	R	1,44	R	1,54	R	1,55	R	1,18	R	1,18	R
g139	1,54	R	1,54	R	1,31	R	1,31	R	1,55	R	1,54	R
g14	1,52	R	1,52	R	1,4	R	1,35	R	1,45	R	1,44	R
g143	1,44	R	1,43	R	1,71	S	1,69	S	1,77	S	1,77	S
g145	1,55	R	1,49	R	1,52	R	1,46	R	1,47	R	1,46	R
g146	1,6	S	1,6	S	1,54	R	1,54	R	1,2	R	1,19	R
g147	1,5	R	1,5	R	1,41	R	1,41	R	1,83	S	1,83	S
g15	1,62	S	1,54	R	1,45	R	1,45	R	1,54	R	1,54	R
g18	1,44	R	1,45	R	1,67	S	1,59	R	1,49	R	1,49	R
g2	1,37	R	1,51	R	1,59	R	1,6	S	1,24	R	1,23	R
g21	1,4	R	1,4	R	1,86	S	1,4	R	1,38	R	1,38	R
g23	1,78	S	1,78	S	1,77	S	1,77	S	1,57	R	1,56	R
g24	1,61	S	1,61	S	1,39	R	1,4	R	1,13	R	1,13	R
g25	1,68	S	1,69	S	1,44	R	1,46	R	1,48	R	1,47	R
g26	1,81	S	1,8	S	1,75	S	1,73	S	1,52	R	1,51	R
g29	1,94	S	1,94	S	1,91	S	1,91	S	1,89	S	1,89	S
g3	1,48	R	1,52	R	1,35	R	1,4	R	1,47	R	1,46	R
g30	1,62	S	1,6	S	1,55	R	1,51	R	1,65	S	1,64	S
g31	1,36	R	1,32	R	1,51	R	1,49	R	1,65	S	1,65	S
g33	1,4	R	1,42	R	1,67	S	1,68	S	1,14	R	1,13	R
g4	1,61	S	1,63	S	1,19	R	1,21	R	1,49	R	1,48	R
g40	1,68	S	1,67	S	1,61	S	1,6	S	1,74	S	1,74	S
g41	1,66	S	1,66	S	1,59	R	1,59	R	1,78	S	1,77	S
g42	1,51	R	1,49	R	1,55	R	1,53	R	1,49	R	1,48	R
g45	1,4	R	1,35	R	1,62	S	1,57	R	1,67	S	1,67	S
g46	1,56	R	1,73	S	1,61	S	1,6	S	1,35	R	1,34	R
g47	1,42	R	1,42	R	1,58	R	1,58	R	1,5	R	1,49	R
g5	1,3	R	1,3	R	1,49	R	1,49	R	1,45	R	1,45	R
g51	1,69	S	1,57	R	1,59	R	1,55	R	1,53	R	1,52	R
g52	1,67	S	1,67	S	1,6	S	1,6	S	1,42	R	1,41	R
g53	1,36	R	1,28	R	1,6	S	1,53	R	1,48	R	1,48	R
g55	1,57	R	1,48	R	1,4	R	1,43	R	1,76	S	1,75	S
g56	1,6	S	1,59	R	1,47	R	1,46	R	1,73	S	1,72	S
g57	1,28	R	1,28	R	1,57	R	1,57	R	1,43	R	1,43	R
g6	1,63	S	1,63	S	1,68	S	1,68	S	1,48	R	1,47	R
g62	1,44	R	1,41	R	1,64	S	1,63	S	1,68	S	1,68	S
g63	1,28	R	1,34	R	1,59	R	1,63	S	1,6	S	1,59	R
g64	1,72	S	1,68	S	1,68	S	1,65	S	1,75	S	1,74	S
g69	1,56	R	1,54	R	1,58	R	1,55	R	1,56	R	1,56	R
g70	1,39	R	1,32	R	1,62	S	1,56	R	1,48	R	1,47	R
g74	1,29	R	1,29	R	1,97	S	1,97	S	1,21	R	1,2	R
g75	1,75	S	1,75	S	1,9	S	1,9	S	1,48	R	1,47	R
g77	1,39	R	1,35	R	1,55	R	1,52	R	1,64	S	1,63	S
g78	1,57	R	1,52	R	1,38	R	1,33	R	1,53	R	1,52	R
g79	1,29	R	1,23	R	1,43	R	1,38	R	1,24	R	1,24	R
g80	1,69	S	1,6	S	1,54	R	1,47	R	1,78	S	1,77	S
g81	1,59	R	1,59	R	1,63	S	1,63	S	1,4	R	1,4	R
g82	1,89	S	1,88	S	1,8	S	1,79	S	1,86	S	1,9	S
g85	1,7	S	1,7	S	1,48	R	1,42	R	1,74	S	1,74	S
g88	1,61	S	1,63	S	1,56	R	1,48	R	1,66	S	1,65	S
g91	1,75	S	1,72	S	1,57	R	1,49	R	1,4	R	1,39	R
g92	1,65	S	1,6	S	1,96	S	1,93	S	1,66	S	1,66	S
g95	1,58	R	1,53	R	1,7	S	1,67	S	1,77	S	1,76	S
g96	1,43	R	1,38	R	1,51	R	1,43	R	1,38	R	1,38	R
g97	1,61	S	1,55	R	1,42	R	1,37	R	1,32	R	1,31	R
g99	1,7	S	1,62	S	1,74	S	1,67	S	1,77	S	1,76	S
