# Supplementary material for: Major Novel QTL for Resistance to Cassava Bacterial Blight Identified through a Multi-Environmental Analysis
Source: Front Plant Sci. 2017 Jul 5;8:1169. doi: 10.3389/fpls.2017.01169 (PMC5496946; doi:10.3389/fpls.2017.01169)
Supplement: Supplementary file 2 [file Table2.PDF]

Novel genetic factors for resistance to cassava bacterial blight detected through a multi-environmental analysis

Johana Carolina Soto Sedano1 jcsotos@unal.edu.co, ORCID ID: 0000-0002-3601-7256, Rubén Eduardo Mora Moreno1, Boby Mathew2 boby.mathew@hotmail.com, Jens Léon2 ulp201@uni-bonn.de, Fabio Andrés Gómez Cano3 fabioandres220@gmail.com Agim Ballvora2 ballvora@uni-bonn.de, Camilo Ernesto López Carrascal1* celopezc@unal.edu.co, telephone number 571- 3165000 ext.11328 *Corresponding autor

1 Manihot Biotec Laboratory, Biology department, Universidad Nacional de Colombia, Bogotá, Colombia.

2 INRES-Plant Breeding University of Bonn, Bonn, Germany.

3 Center for Applied Plant Sciences (CAPS), The Ohio State University, Columbus, USA.

Online Resource 3 Analysis of variance for genotype (g), environment (location, *Xam* strain, season) and genotype x environment among the F1 mapping population. ($p<0.001$)

	Df	Sum Sq	Mean Sq	F value	Pr(>F)
**Rep**	4	1.81	0.45	14.56	8.52e-12 ***
**Environment (E)**	7	9.40	1.34	43.13	<2.2e-16 ***
**Genotype (G)**	116	34.5	0.29	9.56	<2.2e-16 ***
**GxE**	681	62.5	0.09	2.95	<2.2e-16 ***
**Error**	3200	99.6	0.03		
