# Supplementary material for: Major Novel QTL for Resistance to Cassava Bacterial Blight Identified through a Multi-Environmental Analysis
Source: Front Plant Sci. 2017 Jul 5;8:1169. doi: 10.3389/fpls.2017.01169 (PMC5496946; doi:10.3389/fpls.2017.01169)
Supplement: Supplementary file 3 [file Table3.PDF]

Johana Carolina Soto Sedano1 jcsotos@unal.edu.co, ORCID ID: 0000-0002-3601-7256, Rubén Eduardo Mora Moreno1, Boby Mathew2 boby.mathew@hotmail.com, Jens Léon2 ulp201@uni-bonn.de, Fabio Andrés Gómez Cano3 fabioandres220@gmail.com Agim Ballvora2 ballvora@uni-bonn.de, Camilo Ernesto López Carrascal1* celopezc@unal.edu.co, telephone number 571- 3165000 ext.11328
*Corresponding autor
1 Manihot Biotec Laboratory, Biology department, Universidad Nacional de Colombia, Bogotá, Colombia.
2 INRES-Plant Breeding University of Bonn, Bonn, Germany.
3 Center for Applied Plant Sciences (CAPS), The Ohio State University, Columbus, USA.

Online Resource 4 Repertoire of candidate defense-related genes identified in QTLs intervals. CBB resistance QTLs with its positions in the cassava genetic map and in the cassava genome v4.1 and v.6.1. Candidate defense-related genes identified in QTLs intervals with its functional annotation.

QTL	candidate genes within QTLS intervals	Functional annotation Phytozome.com		
QAR318R-5				**KINASAS**<br>**IRG**
QAR318RD-3	Manes.18G090600	PTHR13690:SF86 - TRANSCRIPTION FACTOR VIP1		
	Manes.18G091700	PTHR21654:SF9 - TRIHELIX TRANSCRIPTION FACTOR GT-2		
	Manes.18G091800	PF00892 - EamA-like transporter family (EamA)		
	Manes.08G041000	PTHR11413 - CYSTATIN FAMILY MEMBER		
	**Manes.08G041100**	**PTHR33830:SF3 - DEFENSIN-LIKE PROTEIN 122-RELATED**	DEFENSIN-LIKE PROTEIN 122-RELATED	
	Manes.08G041200	PF07333 - S locus-related glycoprotein 1 binding pollen coat protein (SLR1-BP) (SLR1-BP)		
	Manes.08G041300	PTHR20981 - 60S RIBOSOMAL PROTEIN L21		
QAR681R-17	Manes.08G041400	PTHR21495:SF59 - DIRIGENT PROTEIN 20-RELATED		
	Manes.08G041500	no annotated		
	Manes.08G040900	PTHR12121:SF34 - LD39302P		
	Manes.08G041600	no annotated		
	Manes.08G043500	PF08879 - WRC (WRC)		
	Manes.10G137100	Receptor protein-tyrosine kinase / Receptor protein tyrosine kinase	LRR-Kinase	
QAR681R-19				
	Manes.11G020200	PTHR31602:SF5 - GROWTH-REGULATING FACTOR 5		
QAR681D-14	Manes.11G097300	PTHR13832:SF237 - PROTEIN PHOSPHATASE 2C 46-RELATED		
	Manes.11G097000	KOG4526 - Predicted membrane protein		
QAR681D-19	Manes.10G090800	PTHR23042:SF61 - TRANSCRIPTION FACTOR BHLH18-RELATED		
	Manes.10G091000	PF02201 - SWIB/MDM2 domain (SWIB)		
	**Manes.10G091100**	**no annotated**	**ND**	
	Manes.10G091200	no annotated		
	Manes.10G091300	PTHR12066 - TELOMERASE REVERSE TRANSCRIPTASE		
	**Manes.10G091600**	**PTHR23155//PTHR23155:SF562 - LEUCINE-RICH REPEAT-CONTAINING PROTEIN**	NB-ARC domain	
	**Manes.10G091700**	**PTHR23155//PTHR23155:SF562 - LEUCINE-RICH REPEAT-CONTAINING PROTEIN**	NB-ARC-LRR domain	
	Manes.10G091800	no annotated		
	Manes.10G091900	PTHR16254:SF6 - K(+) EFFLUX ANTIPORTER 3, CHLOROPLASTIC		
	Manes.10G092000	PTHR13812//PTHR13812:SF20 - ORNITHINE CYCLODEAMINASE-RELATED		
	Manes.10G092200	no annotated		
	Manes.10G092300	PTHR32285:SF23 - PROTEIN TRICHOME BIREFRINGENCE-LIKE 12		
	**Manes.10G092400**	**PTHR23155//PTHR23155:SF563 - LEUCINE-RICH REPEAT-CONTAINING PROTEIN**	NB-ARC domain	
	**Manes.10G092600**	**PTHR23155//PTHR23155:SF563 - LEUCINE-RICH REPEAT-CONTAINING PROTEIN**	NB-ARC domain	
	Manes.10G091400	PF03137 - Organic Anion Transporter Polypeptide (OATP) family (OATP)		
	**Manes.10G091500**	**PTHR23155//PTHR23155:SF562 - LEUCINE-RICH REPEAT-CONTAINING PROTEIN**	NB-ARC domain	
	Manes.10G092100	PTHR12170:SF3 - REQUIRED FOR MEIOTIC NUCLEAR DIVISION 5 HOMOLOG B (S. CEREVISIAE)		
	**Manes.10G092500**	**PTHR23155//PTHR23155:SF563 - LEUCINE-RICH REPEAT-CONTAINING PROTEIN**	NB-ARC domain	
	Manes.10G090900	L-galactose 1-dehydrogenase / L-galDH		
QLV318RD-19	**Manes.03G137600**	**WRKY TRANSCRIPTION FACTOR 20-RELATED**	WRKY DNA -binding domain	
	Manes.10G136000	PTHR23091:SF4 - ARD1 HOMOLOG A, N-ACETYLTRANSFERASE		
	Manes.10G136100	PTHR12385:SF4 - PROTEIN PNS1		
	Manes.10G136200	PF09495 - Protein of unknown function (DUF2462) (DUF2462)		
	Manes.10G136300	PTHR23105:SF5 - 39S RIBOSOMAL PROTEIN L1, MITOCHONDRIAL		
	Manes.10G136500	PTHR30603:SF24 - RNA POLYMERASE SIGMA FACTOR SIGD, CHLOROPLASTIC		
	**Manes.10G135900**	**PF00931//PF13855 - NB-ARC domain (NB-ARC) // Leucine rich repeat (LRR_8)**	NB-ARC-LRR domain	
	Manes.10G136400	PTHR19306:SF1 - STRUCTURAL MAINTENANCE OF CHROMOSOMES PROTEIN 5		
	Manes.13G044000	PTHR10042 - EARLY GROWTH RESPONSE PROTEIN-RELATED		
QLV681RD-6	Manes.13G049000	PTHR23041:SF63 - C3HC4 ZINC FINGER DOMAIN-CONTAINING PROTEIN-RELATED		
	Manes.13G039400	PTHR10795:SF350 - SUBTILISIN-LIKE PROTEASE SDD1		
QLV681R-7	Manes.06G088500	PF05701 - Weak chloroplast movement under blue light (WEMBL)		
	Manes.06G088600	PTHR11673 - TRANSLATION INITIATION FACTOR 5A-RELATED		
	Manes.06G088700	PF01844 - HNH endonuclease (HNH)		
	Manes.06G088900	no annotated		
	Manes.06G089000	no annotated		
	Manes.06G089100	PTHR23213:SF210 - FORMIN-LIKE PROTEIN 15A-RELATED		
	Manes.06G089200	no annotated		
	**Manes.06G089400**	**PF11721 - Protein tyrosine kinase (Pkinase_Tyr) // Di-glucose binding**	**Kinase**	
	Manes.06G089500	no annotated		
	Manes.06G089600	PTHR31311:SF4 - GLYCOSYLTRANSFERASE 3-RELATED		
	Manes.06G089700	no annotated		
	Manes.06G089900	PTHR11654:SF181 - PROTEIN NRT1/ PTR FAMILY 2.8		
	Manes.06G090000	PTHR30231 - DNA POLYMERASE III SUBUNIT EPSILON		
	Manes.06G090200	no annotated		
	Manes.06G090300	PTHR10641:SF537 - F21H2.9 PROTEIN-RELATED		
	Manes.06G090400	PTHR10641:SF537 - F21H2.9 PROTEIN-RELATED		
	Manes.06G090500	PTHR31744:SF7 - NAC DOMAIN-CONTAINING PROTEIN 100-RELATED		
	Manes.06G090600	PF01535//PF13041 - PPR repeat (PPR) // PPR repeat family (PPR_2)		
	Manes.06G090700	KOG3292 - Predicted membrane protein		
	Manes.06G090800	PTHR36315:SF1 - NDH DEPENDENT FLOW 6 PROTEIN		
	Manes.06G090900	PTHR31867:SF22 - EXPANSIN-A8		
	Manes.06G091000	PTHR36315:SF1 - NDH DEPENDENT FLOW 6 PROTEIN		
	Manes.06G091100	PTHR10438:SF263 - THIOREDOXIN-LIKE 1-2, CHLOROPLASTIC		
	Manes.06G091200	no annotated		
	Manes.06G091300	PF01357 - Pollen allergen (Pollen_allerg_1)		
	**Manes.06G091400**	**PTHR21649:SF17 - CHLOROPHYLL A-B BINDING PROTEIN 4, CHLOROPLASTIC**	**Chlorophyll A-B binding protein**	
	Manes.06G091500	PTHR11945:SF156 - MADS-BOX PROTEIN-RELATED		
	Manes.06G091600	PTHR31089:SF4 - CYCLIC DOF FACTOR 2-RELATED		

	**Manes.06G091700**	**no annotated**	**ND**
	Manes.06G091900	PTHR11540//PTHR11540:SF29 - MALATE AND LACTATE DEHYDROGENASE	
	Manes.06G092000	PTHR10270 - SOX TRANSCRIPTION FACTOR	
	Manes.06G092100	PTHR33415:SF3 - EMB514	
	Manes.06G092200	PTHR23012//PTHR23012:SF57 - MEMBRANE ASSOCIATED RING FINGER	
	Manes.06G092300	no annotated	
	Manes.06G092400	PTHR23201:SF21 - GIBBERELLIN-REGULATED PROTEIN 4	
	**Manes.06G088800**	**PTHR24056:SF228 - CYCLIN-DEPENDENT KINASE-LIKE PROTEIN-RELATED**	**Kinase**
	Manes.06G089800	PTHR26402:SF465 - TWO-COMPONENT RESPONSE REGULATOR-LIKE APRR1-RELATED	
	Manes.06G090100	Ribulose-phosphate 3-epimerase / Xylulose phosphate 3-epimerase	
	Manes.06G091800	PTHR22835//PTHR22835:SF267 - ZINC FINGER FYVE DOMAIN CONTAINING PROTEIN	
	Manes.06G089300	PTHR23327:SF9 - ZINC FINGER (C3HC4-TYPE RING FINGER) FAMILY PROTEIN	
QLV681D-4	Manes.07G105900	PTHR23088:SF27 - NITRILASE HOMOLOG 1	
	Manes.07G106000	PF06232 - Embryo-specific protein 3, (ATS3) (ATS3)	
	Manes.07G106100	PTHR18937//PTHR18937:SF221 - STRUCTURAL MAINTENANCE OF CHROMOSOMES SMC FAMILY MEMBER	
	Manes.07G106200	PTHR12170:SF2 - MACROPHAGE ERYTHROBLAST ATTACHER	
	Manes.07G106400	PTHR10484 - HISTONE H4	
	Manes.07G106600	no annotated	
	Manes.07G106700	PTHR19241:SF180 - ABC TRANSPORTER G FAMILY MEMBER 1-RELATED	
	Manes.07G106900	PF10551 - FAR1 DNA-binding domain (FAR1) // SWIM zinc finger (SWIM)	
	**Manes.07G107000**	**PF11721 - Protein tyrosine kinase (Pkinase_Tyr) // Di-glucose binding**	**Protein tyrosine kinase**
	**Manes.07G107100**	**PTHR27003:SF13 - LEUCINE-RICH REPEAT PROTEIN KINASE-LIKE PROTEIN**	LEUCINE-RICH REPEAT PROTEIN KINASE-LIKE PROTEIN
	Manes.07G107200		
	Manes.07G107400		
	Manes.07G107500		
	Manes.07G107700		
	**Manes.07G107900**	**Non-specific protein-tyrosine kinase / Cytoplasmic protein tyrosine kinase**	**Kinase**
	**Manes.07G108000**	**Non-specific protein-tyrosine kinase / Cytoplasmic protein tyrosine kinase**	**Kinase**
	Manes.07G108100	no annotated	
	**Manes.07G108200**	**Non-specific protein-tyrosine kinase / Cytoplasmic protein tyrosine kinase**	**Kinase**
	Manes.07G108300	PTHR13872 - 60S RIBOSOMAL PROTEIN L35	
	**Manes.07G108400**	**Non-specific protein-tyrosine kinase / Cytoplasmic protein tyrosine kinase**	**Kinase**
	Manes.07G108600	no annotated	
	**Manes.07G108700**	**Non-specific protein-tyrosine kinase / Cytoplasmic protein tyrosine kinase**	**Kinase**
	**Manes.07G108800**	**Non-specific protein-tyrosine kinase / Cytoplasmic protein tyrosine kinase**	**Kinase**
	Manes.07G108900	PTHR13872 - 60S RIBOSOMAL PROTEIN L35	
	Manes.07G109100	PTHR10644:SF1 - SPLICING FACTOR 3B SUBUNIT 3	
	Manes.07G109200	PTHR21495:SF51 - DIRIGENT PROTEIN 24-RELATED	
	Manes.07G106300	PTHR12436:SF20 - 80 KDA MCM3-ASSOCIATED PROTEIN	
	Manes.07G106800	PTHR14212 - U4/U6-ASSOCIATED RNA SPLICING FACTOR-RELATED	
	Manes.07G107300	no annotated	
	Manes.07G107600	PTHR24177:SF10 - ANKYRIN REPEAT-CONTAINING PROTEIN	
	**Manes.07G107800**	**PTHR23155//PTHR23155:SF580 - LEUCINE-RICH REPEAT-CONTAINING PROTEIN**	NB-ARC domain
	**Manes.07G108500**	**PTHR27001:SF191 - PROTEIN KINASE APK1A, CHLOROPLASTIC-RELATED**	**Kinase**
	Manes.07G109000	PTHR11614:SF92 - ALPHA/BETA-HYDROLASES SUPERFAMILY PROTEIN-RELATED	
	Manes.07G106500	PTHR13271//PTHR13271:SF11 - UNCHARACTERIZED PUTATIVE METHYLTRANSFERASE	
QGH318-8	Manes.03G063200	PTHR22792:SF64 - LA PROTEIN 1-RELATED	
	Manes.03G141900	PTHR15092:SF22 - TARGET OF EGR1 PROTEIN 1	
	**Manes.03G142000**	**PF05627 - Cleavage site for pathogenic type III effector avirulence factor Avr (AvrRpt-cleavage)**	Cleavage site Avr
	Manes.03G141800	no annotated	
	Manes.03G142100	PTHR22811:SF50 - TRANSMEMBRANE EMP24 DOMAIN-CONTAINING PROTEIN P24BETA2	
	Manes.03G142200	PTHR13301:SF60 - CELLULOSE SYNTHASE-LIKE PROTEIN E1	
QGH318-13	Manes.16G060400	PF12710 - E1-E2 ATPase (E1-E2_ATPase) // Heavy-metal-associated domain (HMA)	
QGH318-19	Manes.01G139500	PTHR23070:SF26 - AAA-TYPE ATPASE LIKE PROTEIN	
	Manes.01G139600	PTHR21148:SF25 - PHOSDUCIN-LIKE PROTEIN 1	
	Manes.01G139700	PTHR23324:SF55 - PHOSPHATIDYLINOSITOL/PHOSPHATIDYLCHOLINE TRANSFER PROTEIN	
	Manes.01G139800	no annotated	
	Manes.01G140000	no annotated	
	Manes.01G140100	PTHR31388:SF3 - PEROXIDASE 36-RELATED	
	Manes.01G139400	3-hydroxyacyl-[acyl-carrier-protein] dehydratase	
	Manes.01G139900	PTHR14091 - WD REPEAT PROTEIN	
QGH681-5	Manes.04G035000	PTHR32295:SF18 - PROTEIN IQ-DOMAIN 14-RELATED	
	Manes.04G035200	PTHR24031:SF292 - ATP-DEPENDENT RNA HELICASE DDX54	
QGH681-10	Manes.14G100900	PTHR23151:SF61 - DIHYDROLIPOAMIDE ACETYL/SUCCINYL-TRANSFERASE-RELATED	
	**Manes.14G101000**	**PTHR10219:SF28 - GLYCOLIPID TRANSFER PROTEIN-RELATED**	
QGH681-2.2	Manes.05G106500	PTHR22891:SF9 - PROTEIN ARGONAUTE 7	
	Manes.05G107600	PTHR11106:SF65 - GANGLIOSIDE INDUCED DIFFERENTIATION ASSOCIATED PROTEIN	
	Manes.05G107700	no annotated	
	**Manes.05G107800**	**ANKYRIN REPEAT AND PROTEIN KINASE DOMAIN-CONTAINING PROTEIN**	**Kinase**
	Manes.05G107900	no annotated	
	Manes.05G108000	no annotated	
	Manes.05G108100	PTHR31062:SF32 - XYLOGLUCAN ENDOTRANSGLUCOSYLASE/HYDROLASE PROTEIN 25-RELATED	
	Manes.05G107500	PTHR33167:SF3 - F16A14.15-RELATED	
