# Supplementary material for: Major Novel QTL for Resistance to Cassava Bacterial Blight Identified through a Multi-Environmental Analysis
Source: Front Plant Sci. 2017 Jul 5;8:1169. doi: 10.3389/fpls.2017.01169 (PMC5496946; doi:10.3389/fpls.2017.01169)

Novel genetic factors for resistance to cassava bacterial blight detected through a multi-environmental analysis

Johana Carolina Soto Sedano1 jcsotos@unal.edu.co, ORCID ID: 0000-0002-3601-7256, Rubén Eduardo Mora Moreno1, Boby Mathew2 boby.mathew@hotmail.com, Jens Léon2 ulp201@uni-bonn.de, Fabio Andrés Gómez Cano3 fabioandres220@gmail.com Agim Ballvora2 ballvora@uni-bonn.de, Camilo Ernesto López Carrascal1* celopezc@unal.edu.co, telephone number 571- 3165000 ext.11328 *Corresponding autor 1 Manihot Biotec Laboratory, Biology department, Universidad Nacional de Colombia, Bogotá, Colombia. 2 INRES-Plant Breeding University of Bonn, Bonn, Germany. 3 Center for Applied Plant Sciences (CAPS), The Ohio State University, Columbus, USA.

Online Resource 1a Distribution of AUDPC values for the F1 mapping population for each environment, Xam strain and season. ar= Arauca; lv= La Vega. In figure it is show the AUDPC value for the parents: TMS= parental TMS30572; CM= parental CM2177-2. Shapiro-W Test α= 0,05, **AUDPC normal distributed.

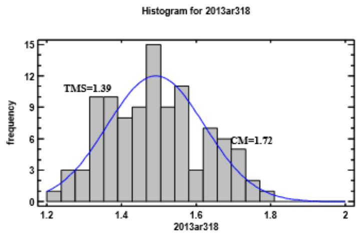

Test	Statistic	P-Value
Shapiro-Wilk W	0.964405	0.051**

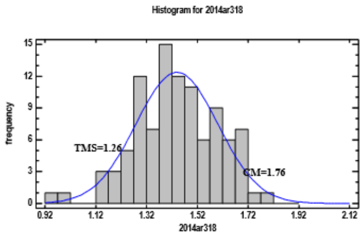

Test	Statistic	P-Value
Shapiro-Wilk W	0.980223	0.538**

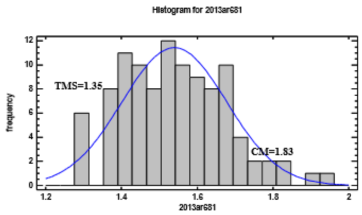

Test	Statistic	P-Value
Shapiro-Wilk W	0.972458	0.192**

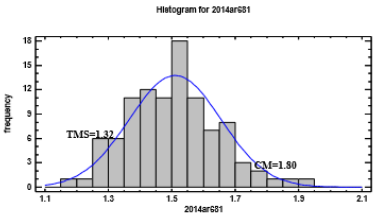

Test	Statistic	P-Value
Shapiro-Wilk W	0.986927	0.866**

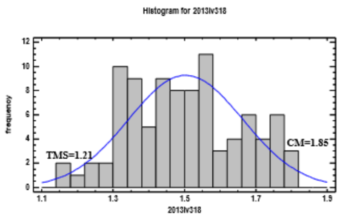

Test	Statistic	P-Value
Shapiro-Wilk W	0.96349	0.054**

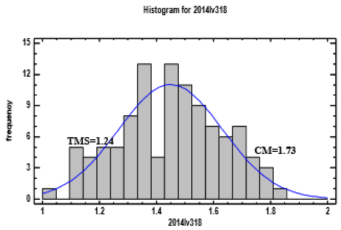

Test	Statistic	P-Value
Shapiro-Wilk W	0.968546	0.096**

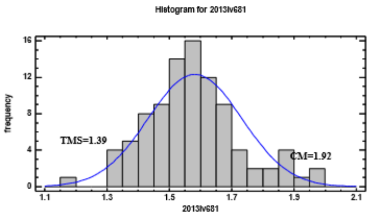

Test	Statistic	P-Value
Shapiro-Wilk W	0.967206	0.102**

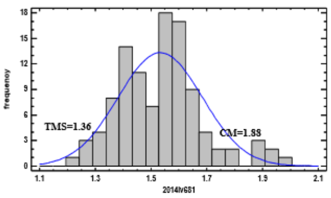

Test	Statistic	P-Value
Shapiro-Wilk W	0.955198	0.006

Online Resource 1b Symptoms scale from 0 to 5 for CBB disease severity. a. 0= no symptoms, b. 1= Necrosis at the inoculation point. c. 2= Stem exudates. d. 3= One or two wilted leaves. e. 4= more than three wilted leaves and f. 5= Plant death.

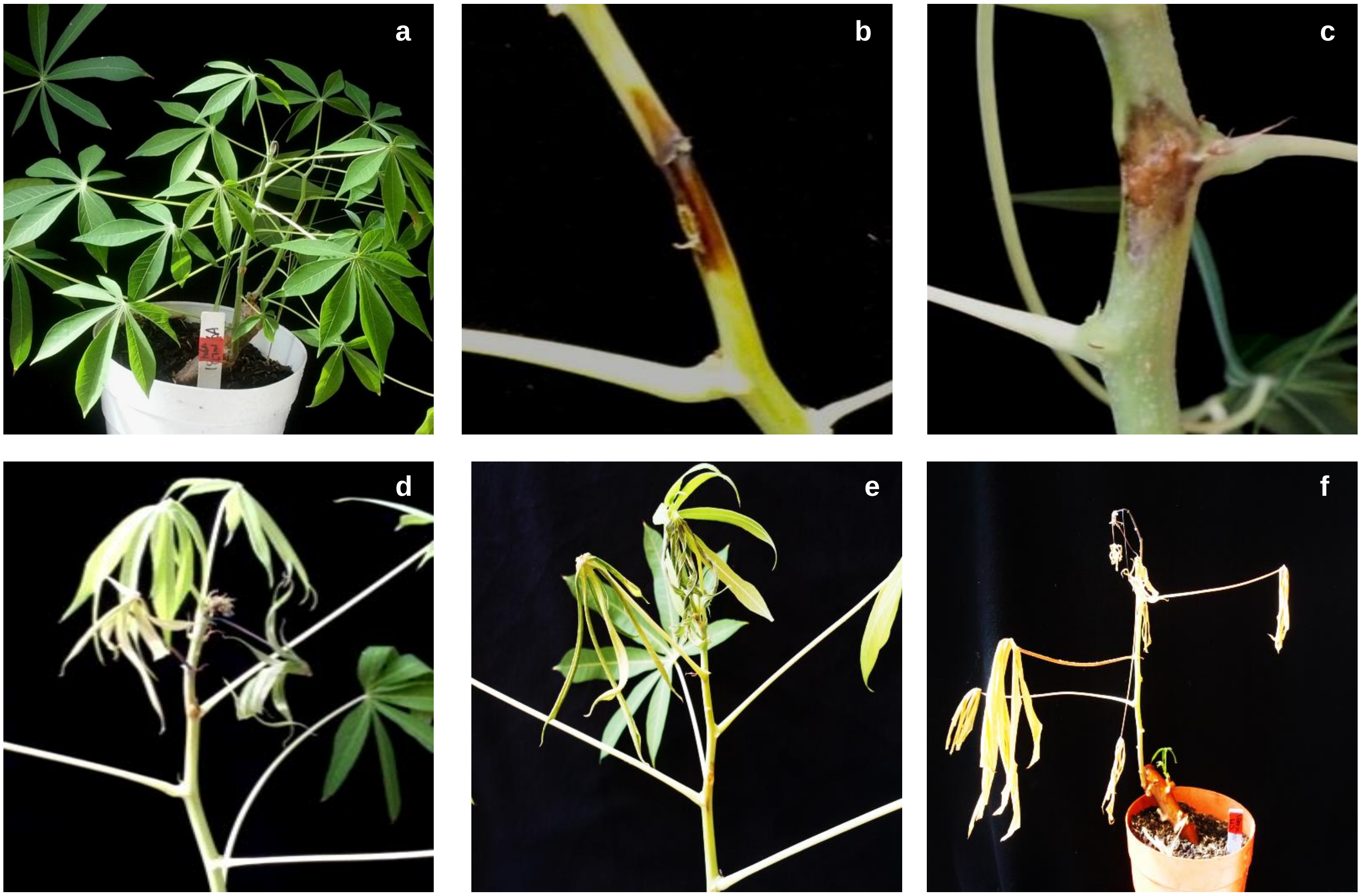

Supplement: Supplementary file 4 [file Image1.PDF]
